# Synthesis of Gallic Acid Analogs as Histamine and Pro-Inflammatory Cytokine Inhibitors for Treatment of Mast Cell-Mediated Allergic Inflammation

**DOI:** 10.3390/molecules22060898

**Published:** 2017-05-29

**Authors:** Xiang Fei, In-Gyu Je, Tae-Yong Shin, Sang-Hyun Kim, Seung-Yong Seo

**Affiliations:** 1 College of Pharmacy and Gachon Institute of Pharmaceutical Sciences, Gachon University, Incheon 21936, Korea; drfei@gachon.ac.kr; 2 Department of Pharmacology, School of Medicine, Kyungpook National University, Daegu 41944, Korea; ingyu23@naver.com; 3 College of Pharmacy, Woosuk University, Jeonbuk 565-701, Korea; tyshin@woosuk.ac.kr

**Keywords:** gallic acid, allergic inflammation, histamine, pro-inflammatory cytokine

## Abstract

Gallic acid (3,4,5-trihydroxybenzoic acid), is a natural product found in various foods and herbs that are well known as powerful antioxidants. Our previous report demonstrated that it inhibits mast cell-derived inflammatory allergic reactions by blocking histamine release and pro-inflammatory cytokine expression. In this report, various amide analogs of gallic acid have been synthesized by introducing different amines through carbodiimide-mediated amide coupling and Pd/C-catalyzed hydrogenation. These compounds showed a modest to high inhibitory effect on histamine release and pro-inflammatory cytokine expression. Among them, the amide bearing (*S*)-phenylglycine methyl ester **3d** was found to be more active than natural gallic acid. Further optimization yielded several (*S*)- and (*R*)-phenylglycine analogs that inhibited histamine release in vitro. Our findings suggest that some gallamides could be used as a treatment for allergic inflammatory diseases.

## 1. Introduction

Gallic acid (3,4,5-trihydroxybenzoic acid), a polyphenol natural product obtained from various herbs, is known to have diverse biological effects such as anti-oxidation, anti-inflammation, and anti-cancer. In a previous research, Shin and coworkers demonstrated that gallic acid inhibits mast cell-mediated inflammatory allergic reactions by blocking histamine release and pro-inflammatory cytokine expression [1].

The prevalence rate of allergic diseases has been rising globally for more than 50 years. Approximately 50% of children are sensitized to common allergens [2]. Allergic inflammation is classified into three phases: early-phase, late-phase, and chronic allergic inflammation. In early-phase reactions, histamine, a major factor in the allergic response, is released from mast cells and induces vasodilation, increases vascular permeability, and recruits leukocytes. Repetitive allergen exposure alters organ function by affecting structural cells and increases production of cytokines resulting in chronic allergic inflammation [3].

Mast cells play key roles in immunoglobulin E (IgE)-mediated allergic reactions through secretion of preformed or newly synthesized mediators such as histamine, lipid-derived mediators, chemokines, cytokines, and growth factors [4]. The signaling pathway of mast cells triggered by antigen cross-linking of IgE bound to FcεRI has been previously described. Stimulation of FcεRI increases degranulation, production of lipid-derived mediators, and expression of cytokines [5]. Therefore, suppression of histamine and pro-inflammatory cytokine release might be an appropriate therapeutic target to reduce allergic inflammation. Human mast cells (HMC-1) are known as an appropriate model for studying allergic reactions characterized by release of histamine and expression of pro-inflammatory cytokines [6,7].

## 2. Results and Discussion

**Figure 1:**
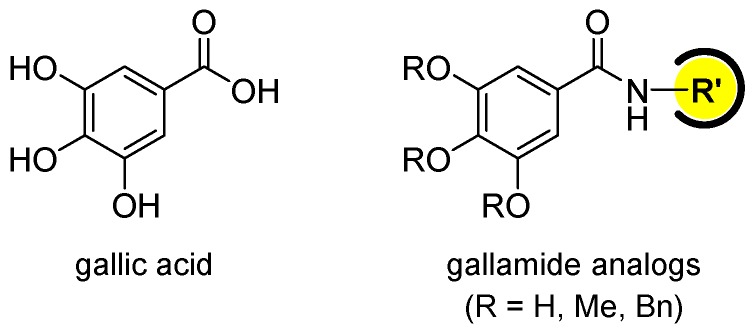
Gallic acid and its amide analogs.

To increase the efficacy of gallic acid, gallic acid analogs were designed and synthesized by the amidation of gallic acid with various amines (Figure 1) [8]. In addition, 3,4,5-trimethoxybenzamides and 3,4,5-trisbenzyloxybenzamides were synthesized in a similar fashion.

**Scheme 1:**
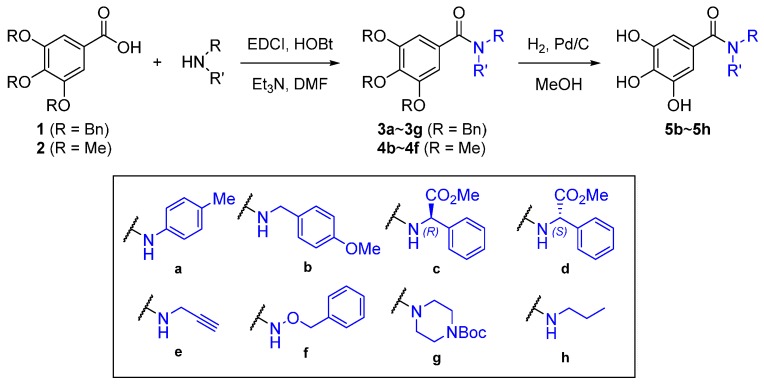
Synthesis of 3,4,5-trisbenzyloxy-, 3,4,5-trimethoxyl- and 3,4,5-trihydroxybenzamides.

Our synthesis of 3,4,5-trihydroxybenzamides commenced with formation of 3,4,5-tris(benzyloxy)benzoic acid amides using some selected amines, followed by hydrogenolysis of all benzyl groups. 1-Ethyl-3-(3-dimethylaminopropyl)carbodiimide (EDCI) was chosen as a carboxyl-activating agent for the amide coupling with amines such as *p*-toluidine, *p*-methoxybenzylamine, (*R*)- and (*S*)-phenylglycine methyl ester, propargyl amine, benzyloxyamine and Boc-piperazine. Thus, we obtained the amides **3a**~**3g** from the respective amines, followed by hydrogenolysis under H_2_, Pd/C to obtain the resulting 3,4,5-trihydroxybenzamides **5b**~**5h** in good yield. Similarly, 3,4,5-trimethoxybenzamides **4b**~**4f** were prepared from 3,4,5-trimethoxybenzoic acid (Scheme 1). Compounds **4a**, **4e** and **5h** were previously reported in the literature [9,10,11,12,13,14].

The release of histamine via degranulation is a key characteristic of activated mast cells, and HMC-1 cells stimulated with phorbol 12-myristate 13-acetate and the calcium ionophore A23187 (PMACI) also released a high level of histamine. Gallic acid, a known inhibitor of histamine release, was used as a positive control. Pretreatment with variously modified compounds (10 μM) differently inhibited the level of histamine released by PMACI-stimulated HMC-1 cells as shown in Table 1 and Table 2. Since the *p*-toluidine analog **3a** showed the weakest inhibition rate (8.9% at 10 µM), neither the trimethoxybenzamide nor trihydroxybenzamide of *p*-toluidine **4a** and **5a** was evaluated further. The gallamides with (*R*)-phenylglycine methyl ester **3c** and **4c** were more potent than (*S*)-enantiomers **3d** and **4d**. Unfortunately, reduction of trisbenzyloxy to trihydoxybenzamides **5c** and **5d** decreased the inhibition rate (24% and 36% at 10 µM). In contrast, *p*-methoxybenzylamine derivatives **3b** and **5b** exhibited a lower inhibition rate than did gallic acid. Trimethoxybenzamide **4b** enhanced the histamine release by HMC-1 cells rather than having an inhibitory effect; consequently, **4b** may trigger an allergic reaction. The *N*-propargylamide analog **3e** and *N*-propylamide **5h**, which was obtained by reduction of **3e**, exhibited a lower histamine inhibition rate (39.4% and 31.2% at 10 µM) than did gallic acid. However, the trimethoxybenzamide analog **4e** showed a slightly increased inhibitory activity (67% at 10 µM) compared with that of gallic acid. Compared with gallic acid, 3,4,5-trihydroxybenzamide analogs did not have an improved effect on histamine inhibition in vitro; however, several compounds such as **3c**, **3d**, **3g**, **4c**, **4d**, **4e**, and **5b** exhibited similar or better inhibitory effects on histamine release.

Cytokines such as tumor necrosis factor (TNF)-α and interleukin (IL)-6 are released by mast cells and are more important in chronic-phase allergic reactions. TNF-α play a key role in immunity by activating NF-κB and regulating immune cells. IL-6 maintains CD4^+^ T cell survival and promotes Th2 modulation; moreover, local accumulation of IL-6 produced by mast cells is known to induce passive cutaneous anaphylaxis (PCA) reaction. Therefore, the inhibition of pro-inflammatory cytokines is also a key strategy to treat allergic inflammation. To determine whether gallic acid and its amide analogs can inhibit inflammation, the expression of pro-inflammatory cytokines such as TNF-α and IL-6 was evaluated by real-time PCR. Some compounds showed different inhibitory effects on the gene expression of TNF-α and IL-6. As shown in Table 3, **3d** and **5d** significantly reduced the gene expression of TNF-α, and five compounds—**3c**, **3d**, **3e**, **3g**, and **5d**—reduced expression of IL-6.

**Scheme 2:**
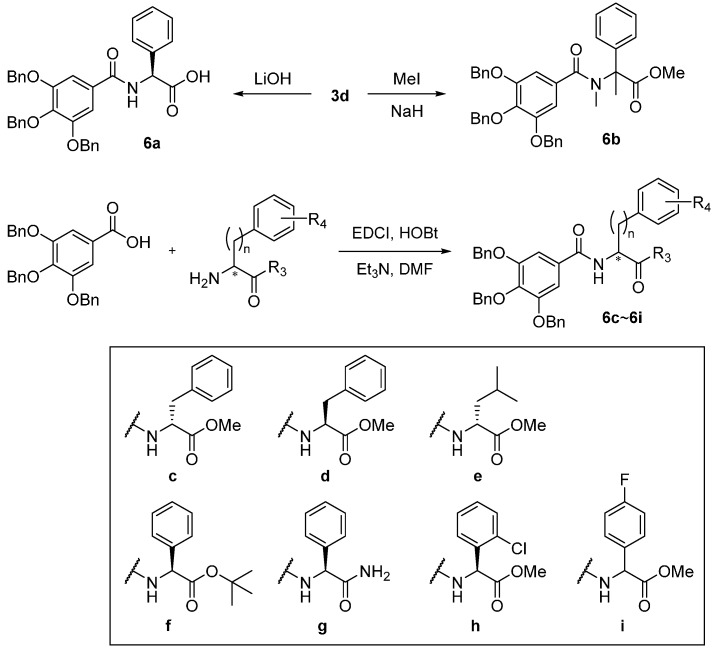
Synthesis of the second-generation gallamide analogs.

Combined with the result of the inhibitory effect on histamine release, **3c** and **3d** significantly inhibited both histamine release and expression of TNF-α and IL-6. Due to the inhibitory effect on both histamine and pro-inflammatory cytokine release, **3c** and **3d** were targeted for more modifications in order to improve their biological activity and drug-like properties. In addition, the analogs derived from **3c** and **3d** could enable us to elucidate structure-activity relationships and modes of action (Scheme 2). Thus, the methyl ester **3d** was hydrolyzed by LiOH in H_2_O/THF to furnish the carboxylic acid **6a**. In a second modification, **3d** was methylated with two equivalents of MeI in the presence of NaH at 0 °C to furnish the tertiary amide **6b**. Various gallamides **6e~6i** were prepared by EDCI-mediated coupling with (*S*)-leucine methyl ester, (*R*)- and (*S*)-phenylalanine methyl ester, (*S*)-phenylalanine *tert*-butyl ester, (*S*)-2-amino-2-phenylacetamide, (*S*)-(+)-2-chloro-phenylglycine methyl ester and (±)-4-fluorophenylglycine methyl ester.

As shown in Table 4, we evaluated the inhibitory activity of the second-generation gallamide analogs at 10 nM on histamine release. We found that **3d** and some derivatives markedly inhibited histamine release even at quite low concentrations. The carboxylic acid **6a** and the dimethylated amide **6b** of **3d** exhibited a similar inhibitory activity on histamine release as did **3d**. Additionally, (*R*)- and (*S*)-phenylalanine methyl esters **6c** and **6d** had a lower inhibition rate than did **3d**. Similar to **4b**, **6f** and **6h** increased the histamine concentration released by HMC-1 cells instead of inhibiting it. The incorporation of an electron-withdrawing group, such as chloro (compound **6h**) and fluoro (compound **6i**), at the aromatic ring of **3d** was not tolerated. Interestingly, leucine amide **6e** showed even better inhibitory activity than the initial compound **3d**.

## 3. Conclusions

It was well established that gallic acid inhibits mast cell-derived inflammatory allergic reactions by blocking histamine release and pro-inflammatory cytokine expression. However, the structure of gallic acid is too small and its hydrophilic polyphenol groups affect metabolism; therefore, it needs to be converted to drug-like compounds. In order to solve these problems, new gallamide analogs were synthesized. Fifteen of 17 compounds could inhibit histamine release; compounds **3c** and **3d** showed greatest inhibitory activity on release of both histamine and pro-inflammatory cytokines such as TNF-α and IL-6. The results give insight into the mechanism by which **3d** exerts anti-allergic and anti-inflammatory activity on mast cells, and suggests that **3d** might be considered as a therapeutic candidate for mast cell-mediated allergic inflammatory diseases.

## 4. Materials and Methods

### 4.1. General Information

All starting materials and reagents were obtained from commercial suppliers and were used without further purification. Air and moisture sensitive reactions were performed under an argon atmosphere. Flash column chromatography was performed using silica gel 60 (230–400 mesh, Merck, Darmstadt, Germany) with the indicated solvents. Thin-layer chromatography was performed using 0.25 mm silica gel plates (Merck, Darmstadt, Germany). ^1^H- (600 MHz) and ^13^C-NMR (150 MHz) spectra were recorded on an AVANCE III System 600 MHz spectrometer (Bruker, Billerica, MA, USA) as solutions in CDCl_3_, DMSO-d_6_ or methanol-*d*_4_. High-resolution mass spectra (HRMS) were obtained on JMS-700 instrument (JEOL, Akishima, Tokyo, Japan) with electrospray ionization. Spectral data of **3a**~**6i** are available in Supplementary Materials.

### 4.2. Synthesis of Gallamide Analogs

*3,4,5-Tris(benzyloxy)-N-p-tolylbenzamide* (**3a**). To a DMF solution (2 mL) of 3,4,5-tris(benzyloxy)benzoic acid (110 mg, 0.25 mmol), *p*-toluidine (32 mg, 0.3 mmol), EDCI (60 mg, 0.4 mmol), HOBt (4 mg, 30 μmol) and TEA (87 μL, 0.50 mmol) were added. After stirring overnight, the reaction mixture was diluted with ethyl acetate and washed with water and brine, dried over MgSO_4_ and concentrated under reduced pressure. The residue was purified by flash column chromatography on silica gel (ethyl acetate/*n*-hexane = 1:2) to generate pure **3a** (111 mg, 80%). ^1^H-NMR (CDCl_3_) δ 7.46 (d, *J* = 8.3 Hz, 2H), 7.44–7.32 (m, 12H), 7.29–7.25 (m, 5H), 7.17 (d, *J* = 8.1 Hz, 3H), 7.12 (s, 2H), 5.15 (s, 4H), 5.12 (s, 2H), 2.34 (s, 3H); ^13^C-NMR (CDCl_3_) δ 165.41, 152.95, 141.52, 137.50, 136.74, 135.48, 134.32, 130.59, 129.71, 128.72, 128.70, 128.35, 128.20, 128.12, 127.69, 120.29, 107.13, 75.32, 71.57, 21.05; HRMS (EI) *m*/*z* calcd. for 529.2253; found 529.2252.

*3,4,5-Tris(benzyloxy)-N-(4-methoxybenzyl)benzamide* (**3b**). To a DMF solution (2 mL) of 3,4,5-tris(benzyloxy)benzoic acid (110 mg, 0.25 mmol), 4-methoxybenzylamine (56 mg, 0.28 mmol), EDCI (115 mg, 0.60 mmol), HOBt (4.0 mg, 30 μmol) and TEA (90 μL, 0.50 mmol) were added. After stirring overnight, the reaction mixture was diluted with ethyl acetate and washed with water and brine, dried over MgSO_4_ and concentrated under reduced pressure. The residue was purified by flash column chromatography on silica gel (ethyl acetate/*n*-hexane = 1:3) to generate pure **3b** (98 mg, 70%). ^1^H-NMR (CDCl_3_) δ 7.21~7.41(m, 15H), 7.05 (s, 2H), 6.90 (m, 2H), 5.11 (s, 4H), 5.08 (s,2H), 4.53 (d, 2H), 3.81 (s, 3H); ^13^C-NMR (CDCl_3_) δ 136.6, 129.3, 128.5, 128.5, 128.2, 128.0, 127.5, 114.1,107.0, 71.5, 29.7; HRMS (EI) *m*/*z* calcd. for 559.2359; found 559.2361.

*(R)-Methyl 2-phenyl-2-(3,4,5-tris(benzyloxy)benzamido)acetate* (**3c**). To a DMF solution (2 mL) of 3,4,5-tris(benzyloxy)benzoic acid (110 mg, 0.25 mmol), (*R*)-2-Phenylglycine methyl ester hydrochloride (61 mg, 0.3 mmol), EDCI (59 mg, 0.4 mmol), HOBt (3.8 mg, 30 μmol) and TEA (90 μL, 0.50 mmol) were added. After stirring overnight, the reaction mixture was diluted with ethyl acetate and washed with water and brine, dried over MgSO_4_ and concentrated under reduced pressure. The residue was purified by flash column chromatography on silica gel (ethyl acetate/*n*-hexane = 1:3) to yield pure **3c** (115 mg, 78%). ^1^H-NMR (CDCl_3_) δ 7.42–7.23 (m, 23H), 7.11 (s, 2H), 6.98 (d, *J* = 6.9 Hz, 1H), 5.70 (d, *J* = 6.9 Hz, 1H), 5.11 (s, 4H), 5.09 (s, 2H), 3.77 (s, 3H); ^13^C-NMR (CDCl_3_) δ 171.83, 166.43, 153.10, 141.92, 137.71, 136.97, 136.78, 129.37, 129.20, 128.98, 128.92, 128.89, 128.54, 128.37, 128.31, 127.92, 127.69, 107.50, 75.49, 71.79, 57.25, 53.28; HRMS (EI) *m*/*z* calcd. for 587.2308; found 587.2307.

*(S)-Methyl 2-phenyl-2-(3,4,5-tris(benzyloxy)benzamido)acetate* (**3d**). To a DMF solution (2 mL) of 3,4,5-tris(benzyloxy)benzoic acid (110 mg, 0.25 mmol), (*S*)-2-phenylglycine methyl ester hydrochloride (56 mg, 0.28 mmol), EDCI (115 mg, 0.60 mmol), HOBt (4.0 mg, 30 μmol) and TEA (90 μL, 0.50 mmol) were added. After stirring for 3 h, the reaction mixture was diluted with ethyl acetate and washed with water and brine, dried over MgSO_4_ and concentrated under reduced pressure. The residue was purified by flash column chromatography on silica gel (ethyl acetate/*n*-hexane = 1:3) to yield pure **3d** (127 mg, 86%). ^1^H-NMR (CDCl_3_) δ 7.41–7.32 (m, 17H), 7.27–7.22 (m, 3H), 7.12 (s, 2H), 7.06 (d, *J* = 6.8 Hz, 1H), 5.69 (d, *J* = 6.9 Hz, 1H), 5.08 (s, 2H), 5.08 (s, 4H), 3.75 (s, 3H); ^13^C-NMR (CDCl_3_) δ 171.59, 166.21, 152.83, 141.64, 137.48, 136.73, 136.52, 129.10, 128.91, 128.71, 128.66, 128.63, 128.28, 128.11, 128.05, 127.67, 127.48, 107.23, 75.23, 71.50, 57.03, 53.01; HRMS (EI) *m*/*z* calcd. for 587.2308; found 587.2307.

*3,4,5-Tris(benzyloxy)-N-(prop-2-yn-1-yl)benzamide* (**3e**). To a DMF solution (4 mL) of 3,4,5-tris-(benzyloxy)benzoic acid (220 mg, 0.50 mmol), propargylamine (33 mg, 0.60 mmol), EDCI (115 mg, 0.60 mmol), HOBt (7.0 mg, 50 μmol) and TEA (90 μL, 0.50 mmol) were added. After stirring for 24 h, the reaction mixture was diluted with ethyl acetate and washed with water and brine, dried over MgSO_4_ and concentrated under reduced pressure. The residue was purified by flash column chromatography on silica gel (ethyl acetate/*n*-hexane = 1:3) to produce the amide **3e** (212 mg, 89%). ^1^H-NMR (CDCl_3_) δ 7.41–7.21 (m, 15H), 7.08 (s, 2H), 6.40 (s, 1H), 5.07 (s, 2H), 5.06 (s, 4H), 4.17 (dd, *J* = 5.2, 2.5 Hz, 2H), 2.24 (t, *J* = 2.5 Hz, 1H); ^13^C-NMR (CDCl_3_) δ 166.90, 152.87, 141.48, 137.46, 136.68, 129.22, 128.65, 128.29, 128.15, 128.07, 127.64, 107.05, 79.63, 75.24, 71.92, 71.46, 29.94; HRMS (EI) *m*/*z* calcd. for 477.1940; found 477.1942.

*N,3,4,5-Tetrakis(benzyloxy)benzamide* (**3f**). To a DMF solution (2 mL) of 3,4,5-tris(benzyloxy)benzoic acid (1) (110 mg, 0.25 mmol), *O*-benzylhydroxylamine hydrochloride (48 mg, 0.3 mmol), EDCI (58 mg, 0.3 mmol), HOBt (4 mg, 30 μmol) and TEA (90 μL, 0.50 mmol) were added. After stirring overnight, the reaction mixture was diluted with ethyl acetate and washed with water and brine, dried over MgSO_4_ and concentrated under reduced pressure. The residue was purified by flash column chromatography on silica gel (ethyl acetate/*n*-hexane = 1:2) to generate pure **3f** (102 mg, 74%). ^1^H-NMR (CDCl_3_) δ 7.42–7.20 (m, 21H), 6.95 (s, 2H), 5.06 (s, 2H), 5.03 (s, 4H), 4.97 (s, 2H); ^13^C-NMR (CDCl_3_) δ 166.20, 152.80, 141.56, 137.35, 136.51, 135.30, 129.39, 128.84, 128.66, 128.56, 128.22, 128.07, 128.00, 127.54, 127.11, 106.83, 78.35, 75.17, 71.27; HRMS (EI) *m*/*z* calcd. for 545.2202; found 545.2205.

*tert-Butyl 4-(3,4,5-tris(benzyloxy)benzoyl)piperazine-1-carboxylate* (**3g**). To a DMF solution (2 mL) of 3,4,5-tris(benzyloxy)benzoic acid (110 mg, 0.25 mmol), 1-Boc-piperazine (56 mg, 0.3 mmol), EDCI (115 mg, 0.60 mmol), HOBt (5.0 mg, 40 μmol) and TEA (90 μL, 0.50 mmol) were added. After stirring overnight, the reaction mixture was diluted with ethyl acetate and washed with water and brine, dried over MgSO_4_ and concentrated under reduced pressure. The residue was purified by flash column chromatography on silica gel (ethyl acetate/*n*-hexane = 1:3) to generate pure **3g** (103 mg, 68%). ^1^H-NMR (CDCl_3_) δ 7.44–7.25 (m, 15H), 6.65 (s, 2H), 5.11 (s, 4H), 5.10 (s, 2H), 3.91–2.81 (m, 8H), 1.47 (s, 9H); ^13^C-NMR (CDCl_3_) δ 170.07, 154.52, 152.68, 139.79, 137.55, 136.70, 130.42, 128.60, 128.26, 128.23, 128.03, 127.98, 127.38, 107.24, 80.29, 75.23, 71.18, 28.43; HRMS (EI) *m*/*z* calcd. for 608.2886; found 608.2888.

*3,4,5-Trihydroxy-N-(4-methoxybenzyl)benzamide* (**4b**). To a CH_2_Cl_2_ solution (3 mL) of 3,4,5-trimethoxybenzoic acid (100 mg, 0.471 mmol), 4-Methoxybenzylamine (78 mg, 0.566 mmol), EDCI (117 mg, 0.613 mmol), HOBt (70 mg, 0.46 mmol) and TEA (0.31 mL, 1.78 mmol) were added. After stirring overnight, the reaction mixture was diluted with ethyl acetate and washed with water and brine, dried over MgSO_4_ and concentrated under reduced pressure. The residue was purified by flash column chromatography on silica gel (ethyl acetate/*n*-hexane = 2:1) to generate 85 mg of **4b** (yield 54%).^1^H-NMR (CDCl_3_) δ 7.23 (d, *J* = 8.6 Hz, 2H), 7.01 (s, 2H), 6.83 (d, *J* = 8.6 Hz, 2H), 6.67 (t, *J* = 5.1 Hz, 1H), 4.51 (d, *J* = 5.7 Hz, 2H), 3.84 (s, 3H), 3.82 (s, 6H), 3.77 (s, 3H); ^13^C-NMR (CDCl_3_) δ 167.26, 159.31, 153.39, 141.09, 130.61, 130.11, 129.54, 114.34, 104.66, 61.16, 56.52, 55.57, 43.94.; HRMS (EI) *m*/*z* calcd. for 289.0950; found 289.0948.

*(R)-Methyl 2-phenyl-2-(3,4,5-trimethoxybenzamido)acetate* (**4c**). To a CH_2_Cl_2_ solution (3 mL) of 3,4,5-trimethoxybenzoic acid (100 mg, 0.471 mmol), (*R*)-(−)-2-phenylglycine methyl ester hydrochloride (114 mg, 0.56 mmol), EDCI (117 mg, 0.613 mmol), HOBt (70 mg, 0.46 mmol) and TEA (0.24 mL, 1.41 mmol) were added. After stirring overnight, the reaction mixture was diluted with CH_2_Cl_2_ and washed with water and brine, dried over MgSO_4_ and concentrated under reduced pressure. The residue was purified by flash column chromatography on silica gel (ethyl acetate/*n*-hexane = 1:1) to generate 85 mg of **4c** (yield 50%). ^1^H-NMR (CDCl_3_) δ 7.46–7.33 (m, 5H), 7.13 (d, *J* = 6.8 Hz, 1H), 7.05 (s, 2H), 5.74 (d, *J* = 6.9 Hz, 1H), 3.89 (s, 6H), 3.88 (s, 3H), 3.78 (s, 3H); ^13^C-NMR (CDCl_3_) δ 171.69, 166.37, 153.30, 141.40, 136.57, 129.15, 129.02, 128.75, 127.51, 104.72, 61.02, 57.10, 56.47, 53.06; HRMS (EI) *m*/*z* calcd. for 359.1369; found 359.1370.

*(S)-Methyl 2-phenyl-2-(3,4,5-trimethoxybenzamido)acetate* (**4d**). To a CH_2_Cl_2_ solution (3 mL) of 3,4,5-trimethoxybenzoic acid (100 mg, 0.471 mmol), (*S*)-(−)-2-phenylglycine methyl ester hydrochloride (110 mg, 0.66 mmol), EDCI (210 mg, 1.09 mmol), HOBt (70 mg, 0.46 mmol) and TEA (0.24 mL, 1.41 mmol) were added. After stirring overnight, the reaction mixture was diluted with CH_2_Cl_2_ and washed with water and brine, dried over MgSO_4_ and concentrated under reduced pressure. The residue was purified by flash column chromatography on silica gel (ethyl acetate/*n*-hexane = 1:1) to produce 90 mg of **4d** (yield 53%). ^1^H-NMR (CDCl_3_) δ 7.43–7.40 (m, 2H), 7.37–7.30 (m, 3H), 7.19 (d, *J* = 6.9 Hz, 1H), 7.04 (s, 2H), 5.72 (d, *J* = 6.9 Hz, 1H), 3.86 (s, 9H), 3.76 (s, 3H); ^13^C-NMR (CDCl_3_) δ 171.65, 166.35, 153.24, 141.33, 136.51, 129.08, 128.95, 128.69, 127.50, 104.69, 60.96, 57.07, 56.40, 52.99; HRMS (EI) *m*/*z* calcd. for 359.1369; found 359.1370.

*3,4,5-Trimethoxy-N-(prop-2-yn-1-yl)benzamide* (**4e**). To a CH_2_Cl_2_ solution (3 mL) of 3,4,5-trimethoxybenzoic acid (100 mg, 0.471 mmol), propargylamine (31 mg, 0.56 mmol), EDCI (250 mg, 1.32 mmol), HOBt (30 mg, 0.20 mmol) and TEA (0.3 mL, 1.71 mmol) were added. After stirring overnight, the reaction mixture was diluted with CH_2_Cl_2_ and washed with water and brine, dried over MgSO_4_ and concentrated under reduced pressure. The residue was purified by flash column chromatography on silica gel (ethyl acetate/*n*-hexane = 1:1) to generate 88 mg of **4e** (yield 52%). ^1^H- NMR (CDCl_3_) δ 7.01 (s, 2H), 6.40 (s, 1H), 4.23 (dd, *J* = 5.3, 2.6 Hz, 2H), 3.88 (s, 6H), 3.87 (s, 3H), 2.28 (s, 1H); ^13^C-NMR (CDCl_3_) δ 166.81, 153.11, 141.02, 129.04, 104.35, 79.40, 71.77, 60.83, 56.23, 56.11, 29.78; HRMS (EI) *m*/*z* calcd. for 317.1263; found 317.1265.

*N-(benzyloxy)-3,4,5-trimethoxybenzamide* (**4f**). To a CH_2_Cl_2_ solution (3 mL) of 3,4,5-trimethoxybenzoic acid (100 mg, 0.471 mmol), *O*-benzylhydroxylamine hydrochloride (83 mg, 0.52 mmol), EDCI (253 mg, 1.32 mmol), HOBt (24 mg, 0.15 mmol) and TEA (0.3 mL, 1.71 mmol) were added. After stirring overnight, the reaction mixture was diluted with CH_2_Cl_2_ and washed with water and brine, dried over MgSO_4_ and concentrated under reduced pressure. The residue was purified by flash column chromatography on silica gel (ethyl acetate/*n*-hexane = 1:1) to generate 115 mg of **4f** (yield 77%). ^1^H- NMR (CDCl_3_) δ 9.01 (s, 1H), 7.46–7.29 (m, 5H), 6.90 (s, 2H), 5.00 (s, 2H), 3.84 (s, 3H), 3.81 (s, 6H); ^13^C-NMR (CDCl_3_) δ 166.33, 153.31, 141.31, 135.32, 129.46, 128.91, 128.70, 127.26, 104.54, 78.45, 60.99, 56.32; HRMS (EI) *m*/*z* calcd. for 317.1263; found 317.1265.

*3,4,5-Trihydroxy-N-(4-methoxybenzyl)benzamide* (**5b**). A 10 mL round-bottom flask was charged with **3b** (30 mg, 0.05 mmol), MeOH (3 mL), and a Teflon-coated magnetic stirring bar, 10% Pd/C (10 mg) were added, producing a black slurry. The flask was equipped with a hydrogen balloon attached to a stainless steel needle. The slurry was left to stir under hydrogen atmosphere. After 24 h, the hydrogen atmosphere of the flask was purged with nitrogen. The mixture was then filtered through a pad of Celite to give a dark green solution. This solution was concentrated to give **5b** (14 mg, 90%). ^1^H-NMR (CD_3_OD) δ 7.24 (d, *J* = 8.7 Hz, 2H), 6.86 (d, *J* = 8.8 Hz, 4H), 4.44 (s, 2H), 3.76 (s, 3H); ^13^C- NMR (CD_3_OD) δ 170.20, 160.14, 146.50, 137.93, 132.29, 129.58, 126.05, 114.69, 107.69, 55.51, 43.73; HRMS (EI) *m*/*z* calcd. for 289.0950; found 289.0948.

*(R)-Methyl 2-phenyl-2-(3,4,5-trihydroxybenzamido)acetate* (**5c**). A 10 mL round-bottom flask was charged with **3a** (89 mg, 0.15 mmol), MeOH (3 mL), and a Teflon-coated magnetic stirring bar. 10% Pd/C (12 mg) was added to the resulting solution, producing a black slurry. The flask was equipped with a hydrogen balloon attached to a stainless steel needle. The slurry was left to stir under hydrogen atmosphere. After 24 h, the hydrogen atmosphere of the flask was purged with nitrogen. The mixture was then filtered through a pad of Celite to give a dark green solution. This solution was concentrated to give **5c** (43 mg, 88%). ^1^H-NMR (CD_3_OD) δ 7.34~7.43 (m, 5H), 6.89 (s, 2H), 5.63 (s, 1H), 3.71 (s, 3H); ^13^C-NMR (CD_3_OD) δ171.49, 168.75, 145.21, 137.04, 136.23, 128.48, 128.13, 127.51, 123.96, 106.76, 57.30, 51.75; HRMS (EI) *m*/*z* calcd. for 317.0899; found 317.0900.

*(S)-Methyl 2-phenyl-2-(3,4,5-trihydroxybenzamido)acetate* (**5d**). A 10 mL round-bottom flask was charged with **4a** (60 mg, 0.1 mmol), MeOH (3 mL), and a Teflon-coated magnetic stirring bar. 10% Pd/C (5 mg) was added to the resulting solution, producing a black slurry. The flask was equipped with a hydrogen balloon attached to a stainless steel needle. The slurry was left to stir under hydrogen atmosphere. After 24 h, the hydrogen atmosphere of the flask was purged with nitrogen. The mixture was then filtered through a pad of Celite to give a dark green solution. This solution was concentrated to give **5d** (30 mg, 92%) ^1^H-NMR (CD_3_OD) δ 7.28~7.41 (m, 5H), 6.86 (s, 2H), 5.60 (s, 1H), 3.68 (s, 3H); ^13^C-NMR (CD_3_OD) δ 171.50, 168.75, 145.24, 137.07, 136.25, 128.47, 128.12, 127.51, 123.96, 106.76, 57.30, 51.70; HRMS (EI) *m*/*z* calcd. for 317.0899; found 317.0900.

*tert-Butyl 4-(3,4,5-trihydroxybenzoyl)piperazine-1-carboxylate* (**5g**). A 10 mL round-bottom flask was charged with **7a** (40 mg, 0.06 mmol), MeOH (3 mL), and a Teflon-coated magnetic stirring bar. 10% Pd/C (10 mg) was added to the resulting solution, producing a black slurry. The flask was equipped with a hydrogen balloon attached to a stainless steel needle. The slurry was left to stir under hydrogen atmosphere. After 24 h, the hydrogen atmosphere of the flask was purged with nitrogen. The mixture was then filtered through a pad of Celite to give a dark green solution. This solution was concentrated to give **5g** (20 mg, 91%). ^1^H-NMR (CD_3_OD) δ 6.36 (s, 2H), 3.64–3.29 (m, 8H), 1.38 (s, 9H); ^13^C-NMR (CD_3_OD) δ 173.19, 156.18, 146.93, 136.53, 126.39, 107.80, 107.60, 81.60, 28.58; HRMS (EI) *m*/*z* calcd. for 338.1478; found 338.1477.

*3,4,5-Trihydroxy-N-(prop-2-ynyl)benzamide* (**5h**). A 10 mL round-bottom flask was charged with **5a** (20 mg, 0.04 mmol), MeOH (3 mL), and a Teflon-coated magnetic stirring bar. 10% Pd/C (5 mg) was added to the resulting solution, producing a black slurry. The flask was equipped with a hydrogen balloon attached to a stainless steel needle. The slurry was left to stir under hydrogen atmosphere. After 24 h, the hydrogen atmosphere of the flask was purged with nitrogen. The mixture was then filtered through a pad of Celite to give a dark green solution. This solution was concentrated and purified by Biotage^®^ SNAP Ultra C18 reversed phase cartridge ( from 10 to 90% ACN with 0.1% TFA) to give **5h** (6 mg, 67%). ^1^H-NMR (CD_3_OD) δ 6.83 (s, 2H), 3.26 (t, *J* = 7.2 Hz, 2H), 1.59 (dd, *J* = 14.5, 7.3 Hz, 2H), 0.95 (t, *J* = 7.4 Hz, 3H); ^13^C-NMR (CD_3_OD) δ 170.59, 146.62, 137.95, 126.41, 107.73, 58.32, 42.67, 23.79, 18.36, 11.75; HRMS (EI) *m*/*z* calcd. for 211.0845; found 211.0842.

*(S)-2-Phenyl-2-(3,4,5-tris(benzyloxy)benzamido)acetic acid* (**6a**). The **3c** (150 mg, 0.26 mmol) was suspended in THF (3 mL) in a 25-mL round-bottomed flask. In a separate flask, lithium hydroxide (16 mg) was dissolved in deionized water (1 mL). Both mixtures were chilled to 4 °C and combined to form a turbid white mixture. After 1 h of stirring, the mixture had become homogeneous. After 24 h, 1 mL of 1 M HCl was added, and the mixture was allowed to warm to room temperature. Following the addition of brine, the mixture was extracted four times with EtOAc, and the combined organic layers were evaporated. This yielded **6a** as white powder (80 mg, 55%). ^1^H-NMR (CD_3_OD) δ 7.90 (s, 1H), 7.51 (dd, *J* = 8.1, 0.9 Hz, 2H), 7.46–7.44 (m, 3H), 7.40–7.29 (m, 13H), 7.25–7.14 (m, 5H), 5.46 (s, 1H), 5.13 (s, 4H), 5.01 (s, 2H); ^13^C-NMR (CD_3_OD) δ 174.62, 168.22, 154.02, 141.95, 141.24, 138.78, 138.32, 131.00, 129.74, 129.54, 129.33, 129.16, 129.06, 129.02, 128.92, 128.60, 128.36, 107.94, 76.19, 72.28, 61.10; HRMS (EI) *m*/*z* calcd. for 573.2151; found 573.2150.

*Methyl 2-phenyl-2-(3,4,5-tris(benzyloxy)-N-methylbenzamido) propanoate* (**6b**). Under a N_2_ atmosphere, to a solution of (*S*)-methyl 2-phenyl-2-(3,4,5-tris(benzyloxy)benzamido)acetate (200 mg, 0.34 mmol) in DMF (2.5 mL), NaH (20 mg, 60% purity, 0.51 mmol) was added slowly at 0 °C. After 30 min, iodomethane (0.042 mL, 0.48 mmol) was added slowly. The solution was stirred for 10 min at room temperature, after which the reaction was quenched by adding an excess amount of saturated NH_4_Cl aqueous solution, followed by extraction with ethyl acetate. The organic phase was washed with brine and then dried over anhydrous MgSO_4_. After the solution was filtered and the solvent was evaporated under vacuum, the residue was subjected to silica gel column chromatography using 25% EtOAc in hexane to give **6b** (30 mg, 15%). H-NMR (CDCl_3_) δ 7.62–7.51 (m, 2H), 7.46–7.18 (m, 19H), 6.87–6.79 (m, 2H), 5.18–5.07 (m, 6H), 3.66 (s, 3H), 2.38 (s, 3H), 1.96 (s, 3H); ^13^C-NMR (CDCl_3_) δ 172.9, 172.1, 152.6, 140.3, 137.8, 137.5, 136.8, 131.0, 128.6, 128.5, 128.4, 128.3, 128.2, 127.9 (2), 127.4, 108.0, 75.2, 66.9, 52.6, 35.9, 18.6. HRMS (EI) *m*/*z* calcd. for 615.2621; found 615.2619.

*Methyl (3,4,5-tris(benzyloxy)benzoyl)-d-phenylalaninate* (**6c**). To a DMF solution (10 mL) of 3,4,5-tris(benzyloxy)benzoic acid (408 mg, 0.92 mmol), d-phenylalanine methyl ester hydrochloride (200 mg, 0.92 mmol), EDCI (196 mg, 1.02 mmol), HOBt (71 mg, 0.46 mmol) and TEA (0.30 mL, 2.04 mmol) were added. After stirring overnight, the reaction mixture was diluted with ethyl acetate and washed with water and brine, dried over MgSO_4_ and concentrated under reduced pressure. The residue was purified by flash column chromatography on silica gel (ethyl acetate/*n*-hexane = 1:3) to generate pure **6c** (220 mg, 40%). ^1^H-NMR (CDCl_3_) δ 7.25–7.41 (m, 18H), 7.11 (dd, 2H, *J* = 1.8, 7.8 Hz), 7.00 (s, 2H), 6.37 (d, 1H), 5.09 (s, 4H), 5.08 (s, 2H), 5.02–5.05 (m, 1H), 3.78 (s, 3H), 3.23 (qd, 2H, *J* = 7.8, 24.6 Hz); ^13^C- NMR (CDCl_3_) δ 172.0, 166.4, 152.7, 141.4, 137.4, 136.6, 135.8, 129.3, 129.2, 128.6, 128.5, 128.0, 127.9, 127.5, 127.2, 106.8, 75.1, 71.3, 53.5, 52.4, 37.8; HRMS (EI) *m*/*z* calcd. for 601.2464; found 601.2463.

*Methyl (3,4,5-tris(benzyloxy)benzoyl)-l-phenylalaninate* (**6d**). To a DMF solution (10 mL) of 3,4,5-tris(benzyloxy)benzoic acid (517 mg, 1.17 mmol), l-phenylalanine methyl ester hydrochloride (229 mg, 1.06 mmol), EDCI (230 mg, 1.20 mmol), HOBt (80 mg, 0.52 mmol) and TEA (0.37 mL, 2.09 mmol) were added. After stirring overnight, the reaction mixture was diluted with ethyl acetate and washed with water and brine, dried over MgSO_4_ and concentrated under reduced pressure. The residue was purified by flash column chromatography on silica gel (ethyl acetate/*n*-hexane = 1:3) to generate pure **6d** (420 mg, 66%). ^1^H-NMR (CDCl_3_) δ 7.25–7.41 (m, 18H), 7.11 (dd, 2H, *J* = 1.8, 7.8 Hz), 7.00 (s, 2H), 6.37 (d, 1H), 5.09 (s, 4H), 5.08 (s, 2H), 5.02–5.05 (m, 1H), 3.78 (s, 3H), 3.23 (qd, 2H, *J* = 7.8, 24.6 Hz); ^13^C- NMR (CDCl_3_) δ 172.0, 166.4, 152.7, 141.4, 137.4, 136.6, 135.8, 129.3, 129.2, 128.6, 128.5, 128.0, 127.9, 127.5, 127.2, 106.8, 75.1, 71.3, 53.5, 52.4, 37.8; HRMS (EI) *m*/*z* calcd. for 601.2464; found 601.2466.

*Methyl (3,4,5-tris(benzyloxy)benzoyl)leucinate* (**6e**). To a CH_2_Cl_2_ solution (3 mL) of 3,4,5-tris(benzyloxy)benzoic acid (240 mg, 0.54 mmol), l-leucine methyl ester hydrochloride (180 mg, 1.06 mmol), EDCI (190 mg, 1.00 mmol), HOBt (30 mg, 0.19 mmol) and TEA (0.14 mL, 0.85 mmol) were added. After stirring overnight, the reaction mixture was diluted with ethyl acetate and washed with water and brine, dried over MgSO_4_ and concentrated under reduced pressure. The residue was purified by flash column chromatography on silica gel (ethyl acetate/*n*-hexane = 1:5) to generate 120 mg of **6e** (yield 47%). ^1^H-NMR (CDCl_3_) δ 7.44–7.17 (m, 15H), 7.10 (s, 2H), 6.93 (d, *J* = 8.1 Hz, 1H), 4.85–4.79 (m, 1H), 3.74 (s, 3H), 1.77–1.56 (m, 3H), 0.96 (dd, *J* = 6.3, 2.7 Hz, 6H); ^3^C-NMR (CDCl_3_) δ 174.61, 166.67, 152.75, 141.35, 137.56, 136.85, 128.91, 128.62, 128.52, 128.21, 127.98, 127.92, 127.77, 106.89, 75.18, 71.32, 52.48, 51.36, 41.37, 25.06, 23.03, 21.85; HRMS (EI) *m*/*z* calcd. for 567.2421; found 567.2466.

*tert-Butyl (S)-2-phenyl-2-(3,4,5-tris(benzyloxy)benzamido)acetate* (**6f**). To a CH_2_Cl_2_ solution (3 mL) of 3,4,5-tris(benzyloxy)benzoic acid (180 mg, 0.40 mmol), l-Phenylalanine tert-butyl ester hydrochloride (100 mg, 0.41 mmol), EDCI (90 mg, 0.47 mmol), HOBt (30 mg, 0.19 mmol) and TEA (0.14 mL, 0.85 mmol) were added. After stirring overnight, the reaction mixture was diluted with ethyl acetate and washed with water and brine, dried over MgSO_4_ and concentrated under reduced pressure. The residue was purified by flash column chromatography on silica gel (ethyl acetate/*n*-hexane = 1:3) to generate 110 mg of **6f** (yield 45%). ^1^H-NMR (CDCl_3_) δ 7.46–7.27 (m, 19H), 7.21 (t, *J* = 6.1 Hz, 1H), 7.19 (s, 2H), 5.65 (d, *J* = 7.0 Hz, 1H), 5.12 (s, 6H), 1.46 (s, 9H); ^13^C-NMR (CDCl_3_) δ 170.22, 165.96, 152.79, 141.48, 137.49, 137.32, 136.74, 129.18, 128.82, 128.63, 128.58, 128.27, 128.24, 128.05, 127.99, 127.66, 127.26, 107.13, 82.84, 75.18, 71.42, 57.40, 27.90; HRMS (EI) *m*/*z* calcd. for 629.2777; found 629.2774.

*(S)-N-(2-Amino-2-oxo-1-phenylethyl)-3,4,5-tris(benzyloxy)benzamide* (**6g**). To a CH_2_Cl_2_ solution (3 mL) of 3,4,5-tris(benzyloxy)benzoic acid (390 mg, 0.88 mmol), (*S*)-2-amino-2-phenylacetamide (180 mg, 1.06 mmol), EDCI (190 mg, 1.00 mmol), HOBt (70 mg, 0.46 mmol) and TEA (0.31 mL, 1.78 mmol) were added. After stirring overnight, the reaction mixture was diluted with ethyl acetate and washed with water and brine, dried over MgSO_4_ and concentrated under reduced pressure. The residue was purified by flash column chromatography on silica gel (ethyl acetate/*n*-hexane = 1:1) to generate 302 mg of **6g** (yield 57%). ^1^H-NMR (DMSO-d_6_) δ 8.75 (d, 1H, *J* = 7.8 Hz), 7.72 (s, 1H), 7.51 (d, 2H, *J* = 7.2 Hz), 7.46 (d, 4H, *J* = 7.2 Hz), 7.23–7.40 (m, 17H), 5.66 (d, 1H, *J* = 7.8 Hz), 5.17 (s, 4H), 4.99 (s, 2H); ^13^C- NMR (DMSO-d_6_) δ 172.26, 165.79, 152.37, 139.21, 137.36, 129.60, 128.84, 128.69, 128.63, 128.47, 128.33, 128.26, 128.12, 127.99, 127.95, 107.35, 74.65, 70.83, 60.18; HRMS (EI) *m*/*z* calcd. for 572.2311; found 572.2312.

*Methyl 2-(2-chlorophenyl)-2-(3,4,5-tris(benzyloxy)benzamido)acetate* (**6h**). To a CH_2_Cl_2_ solution (3 mL) of 3,4,5-tris(benzyloxy)benzoic acid (286 mg, 0.65 mmol), (*S*)-(+)-2-chlorophenylglycine methyl ester hydrochloride (130 mg, 0.65 mmol), EDCI (137 mg, 0.71 mmol), HOBt (50 mg, 0.33 mmol) and TEA (0.34 mL, 1.9 mmol) were added. After stirring overnight, the reaction mixture was diluted with ethyl acetate and washed with water and brine, dried over MgSO_4_ and concentrated under reduced pressure. The residue was purified by flash column chromatography on silica gel (ethyl acetate/*n*-hexane = 1:3) to generate 30 mg of **6h** (yield 8%). ^1^H-NMR (CDCl_3_) δ 7.46–7.26 (m, 18H), 7.13 (d, *J* = 6.9 Hz, 1H), 7.10 (s, 2H), 6.01 (d, *J* = 7.0 Hz, 1H), 5.10 (d, *J* = 6.6 Hz, 4H), 5.09 (s, 2H), 3.77 (d, *J* = 8.4 Hz, 3H); ^13^C-NMR (CDCl_3_) δ 142.80, 138.02, 124.72, 113.56, 109.35, 108.60, 106.68, 105.53, 102.65, 102.19, 101.79, 100.75, 100.56, 100.54, 100.18, 100.00, 99.94, 99.53, 99.30, 79.13, 47.12, 43.37, 27.29, 25.14; HRMS (EI) *m*/*z* calcd. for 621.1918; found 621.1917.

*Methyl 2-(4-fluorophenyl)-2-(3,4,5-tris(benzyloxy)benzamido)acetate* (**6i**). To a CH_2_Cl_2_ solution (3 mL) of 3,4,5-tris(benzyloxy)benzoic acid (390 mg, 0.88 mmol), methyl amino(4-fluorophenyl)acetate hydrochloride (240 mg, 1.09 mmol), EDCI (310 mg, 1.62 mmol), HOBt (70 mg, 0.52 mmol) and TEA (0.57 mL, 3.2 mmol) were added. After stirring overnight, the reaction mixture was diluted with ethyl acetate and washed with water and brine, dried over MgSO_4_ and concentrated under reduced pressure. The residue was purified by flash column chromatography on silica gel (ethyl acetate/*n*-hexane = 1:3) to generate 320 mg of **6i** (yield 48%). ^1^H-NMR (CDCl_3_) δ 7.46–7.29 (m, 15H), 7.29–7.27 (m, 2H), 7.13–7.08 (m, 2H), 7.08–6.99 (m, 2H), 6.99–6.91 (m, 1H), 5.69 (d, *J* = 6.7 Hz, 1H), 5.16–5.12 (m, 4H), 5.10 (s, 2H), 3.79 (s, 3H); ^13^C-NMR (CDCl_3_) δ 171.37, 166.04, 163.59, 161.95, 152.82, 141.73, 137.35, 136.62, 132.46, 129.12, 129.06, 128.73, 128.59, 128.23, 128.08, 128.01, 127.60, 116.06, 115.92, 107.20, 75.18, 71.52, 56.20, 53.07; HRMS (EI) *m*/*z* calcd. for 605.2214; found 605.2217.

### 4.3. Determination of the Mast Cell Degranulation

#### 4.3.1. Histamine Assay Use PMACl in HMC-1 Cells

To determine the mast cell degranulation, level of histamine in culture media was measured. HMC-1 cells were grown in Iscove’s modified Dulbecco’s medium (GIBCO, Grand Island, NY, USA) supplemented with 10% heat-inactivated fetal bovine serum, 100 U/mL penicillin G, 100 mg/mL streptomycin, and 250 ng/mL amphotericin B at 37 °C in 5% CO_2_ incubator. The passage ranging 4–8 of HMC-1 cells (5 × 10^5^/well in 24-well plates) were pretreated with or without drugs for 1 h and then stimulated with 4 nM of phorbol 12-mystate 13-acetate (PMA, Sigma-Aldrich, St Louis, MO, USA) and 1 μM of calcium ionophore A23187 (CI, Sigma-Aldrich) for 8 h. The cells were separated from the media by centrifugation at 150 *g* for 5 min at 4 °C. To separate histamine from serum and media, 0.1 N HCl and 60% perchloric acid were added. After centrifugation, the supernatant fluid transferred to effendorf tube containing 5 N NaOH, 5 M NaCl, and *n*-butanol and then vortexed. The organic phase was gathered, shaken with 0.1 N HCl and *n*-haptane, and then centrifuged. The histamine in the aqueous phase is assayed using the *o*-phthaldialdehyde spectrofluorometric procedure as previously described [6].

#### 4.3.2. Histamine Assay Use DNP-Human Serum Albumin in RBL-2H3 Cells

To evaluate mast cell degranulation, the level of histamine in culture media was measured. RBL-2H3 cells (5 × 10^5^/well in 24-well plates) were sensitized with anti-DNP IgE (50 ng/mL). After incubation overnight, cells were pretreated with or without compound for 1 h and then stimulated with DNP-HAS (100 ng/mL) for 4 h. The cells were separated from the media by centrifugation at 150 g for 5 min at 4 °C. To separate histamine from serum and media, 0.1 N HCl and 60% perchloric acid were added. After centrifugation, the supernatant fluid was transferred to an Eppendorf tube containing 5 N NaOH, 5 M NaCl, and *n*-butanol, then vortexed. The organic phase was gathered, shaken with 0.1 N HCl and *n*-haptane, then centrifuged. The histamine in the aqueous phase was assayed using the *o*-phthaldialdehyde spectrofluorometric procedure as previously described [6].

### 4.4. RNA Extraction and Real-Time PCR

Prior to isolation of total cellular RNA, HMC-1 cells (5 × 10^5^/well in 24-well plates) were pretreated with or without compound for 1 h and then stimulated with PMA (40 nM) and CI (1 μM) for 1 h. RNAiso Plus reagent (Takara Bio Inc., Shiga, Japan) was used to extract total RNA, in accordance with the manufacturer’s protocol. Complementary DNA (cDNA) was synthesized from 2 μg of total RNA using the Maxime RT-Pre Mix Kit (iNtRON Biotechnology, Daejeon, Korea). Quantitative real-time PCR was carried out using the Thermal Cycler Dice TP850 (Takara Bio Inc.) according to the manufacturer’s protocol. The 25 μL reaction mixture was composed as follows: 1.5 μL of cDNA (150 ng), 1 μL of each of the forward and reverse primers (0.4 μM), 12.5 μL of SYBR Premix Ex Taq (Takara Bio Inc.), and 9 μL of D_2_O [15].

### 4.5. Statistical Analysis

Each data represents the mean ± SE of 3 independent experiments. Statistical analyses were performed using Prism5 (GraphPad Software, San Diego, CA, USA), and treatment effects were analyzed using a one-way ANOVA followed by Dunnett’s test. A value of *p* < 0.05 was used to indicate a significant difference.

## Figures and Tables

**Table 1:** Inhibitory activity of 3,4,5-trisbenzyloxy- and 3,4,5-trihydroxybenzamide analogs (10 µM) on histamine release in HMC-1 cells. 
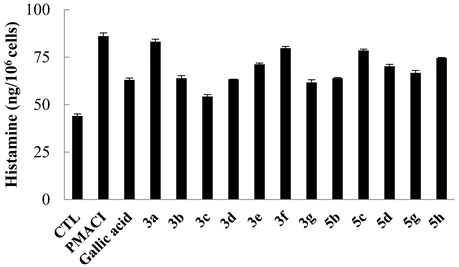

Compound	% Inhibition
Gallic acid	58.9
**3a**	8.9
**3b**	48.3
**3c**	74.3
**3d**	54.9
**3e**	39.4
**3f**	20.9
**3g**	59.9
**5b**	52.1
**5c**	24.3
**5d**	35.8
**5g**	41.9
**5h**	31.2

**Table 2:** Inhibitory activity of 3,4,5-trimethoxylamides (10 µM) on histamine release in HMC-1 cells. 
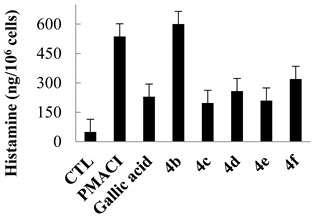

Compound	% Inhibition
Gallic acid	63.0
**4b**	−12.9
**4c**	69.6
**4d**	57.2
**4e**	67.0
**4f**	44.5

**Table 3:** Gene expression of pro-inflammatory cytokines. (Values are related to β-actin) * significant inhibition of cytokines. 
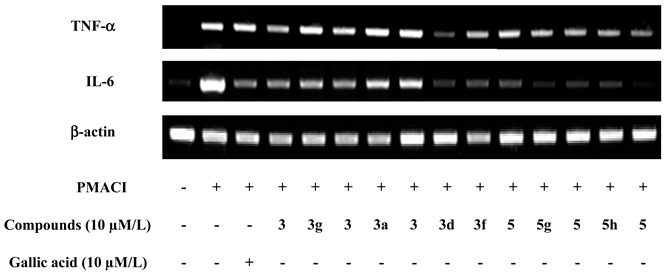

PMACI	Compound (10 µM)	TNF-α	IL-6
-	-	0.35 ± 0.03	0.46 ± 0.06
+	-	0.71 ± 0.06	1.05 ± 0.14
+	**Gallic acid**	0.59 ± 0.02 *	0.67 ± 0.02 *
+	**3a**	0.93 ± 0.22	0.84 ± 0.11
+	**3b**	1.10 ± 0.03	0.81 ± 0.11
+	**3c**	0.88 ± 0.07	0.67 ± 0.05 *
+	**3d**	0.46 ± 0.04 *	0.71 ± 0.08 *
+	**3e**	0.65 ± 0.12	0.59 ± 0.09 *
+	**3f**	0.67 ± 0.12	0.85 ± 0.16
+	**3g**	0.85 ± 0.08	0.68 ± 0.04 *
+	**5b**	0.96 ± 0.22	0.80 ± 0.19
+	**5c**	0.81 ± 0.20	0.81 ± 0.09
+	**5d**	0.50 ± 0.07 *	0.45 ± 0.09 *
+	**5g**	0.92 ± 0.16	0.83 ± 0.08
+	**5h**	0.88 ± 0.11	0.81 ± 0.17

**Table 4:** Inhibitory activity of **3d** and its analogs (10 nM) on histamine release in RBL-2H3 cells. 
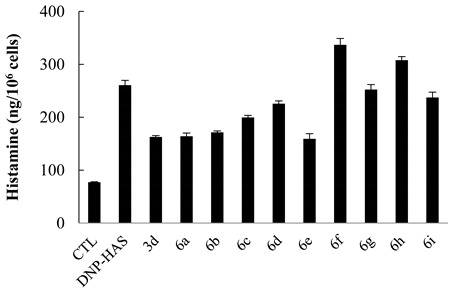

Compound	% Inhibition
**3d**	53
**6a**	52
**6b**	48
**6c**	32
**6d**	18
**6e**	55
**6f**	−42
**6g**	4
**6h**	−26
**6i**	11

## Supplementary Materials

The following are available online, **Figures S1–S52**: Spectral data.
