# Supplementary material for: Synthesis of Gallic Acid Analogs as Histamine and Pro-Inflammatory Cytokine Inhibitors for Treatment of Mast Cell-Mediated Allergic Inflammation
Source: Molecules. 2017 May 29;22(6):898. doi: 10.3390/molecules22060898 (PMC6152652; doi:10.3390/molecules22060898)
Supplement: Supplementary file 1 [file molecules-22-00898-s001.pdf]

Supplementary Materials: Synthesis of Gallic Acid Analogues as Histamine and Pro-Inflammatory Cytokines Inhibitors for Treatment of Mast cell-mediated Allergic Inflammation

Xiang Fei[1], In-Gyu Je[2], Tae-Yong Shin[3], Sang-Hyun Kim[2,*] and Seung-Yong Seo[1,*]

1. General Information

All starting materials and reagents were obtained from commercial suppliers and were used without further purification. Air and moisture sensitive reactions were performed under an argon atmosphere. Flash column chromatography was performed using silica gel 60 (230-400 mesh, Merck) with the indicated solvents. Thin-layer chromatography was performed using 0.25 mm silica gel plates (Merck). ^{1}H and ^{13}C NMR spectra were recorded on a Bruker 600 MHz as solutions in $CDCl_3$, DMSO-d6 or Methanol-d4. High-resolution mass spectra (HRMS) were obtained on a JEOL JMS-700 with electrospray ionization.

2. Spectral data

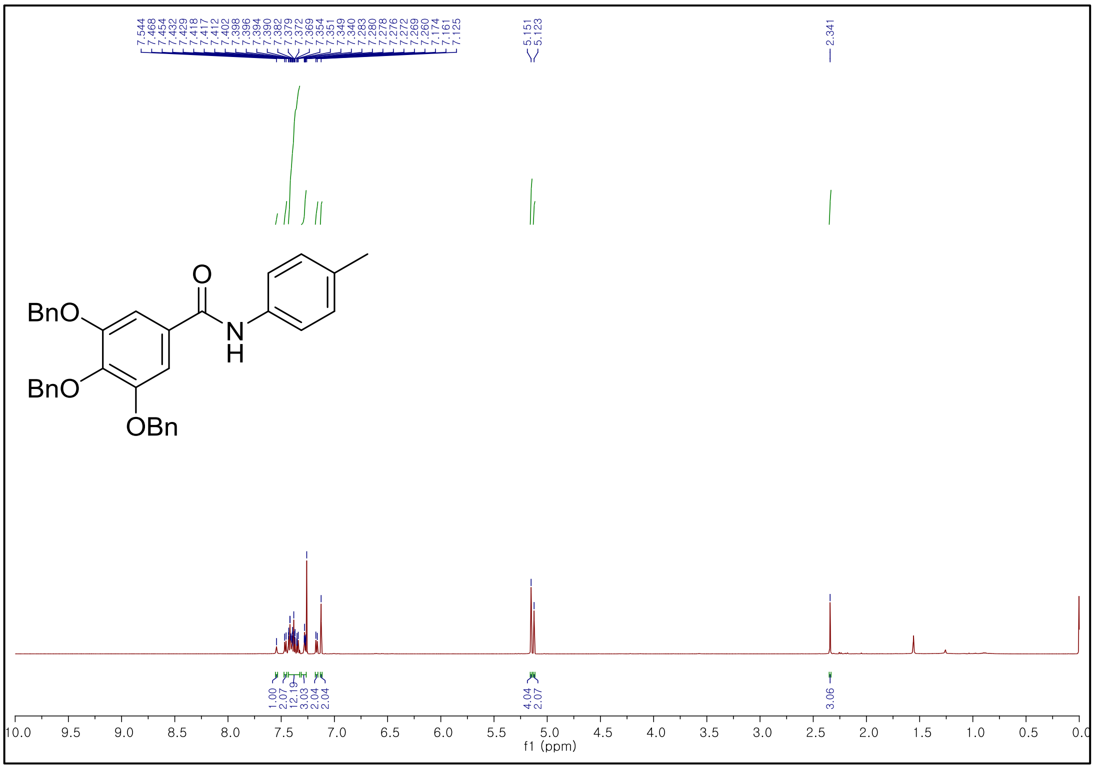

Figure. S1 ^{1}H-NMR (600 MHz, $CDCl_3$) of **3a**

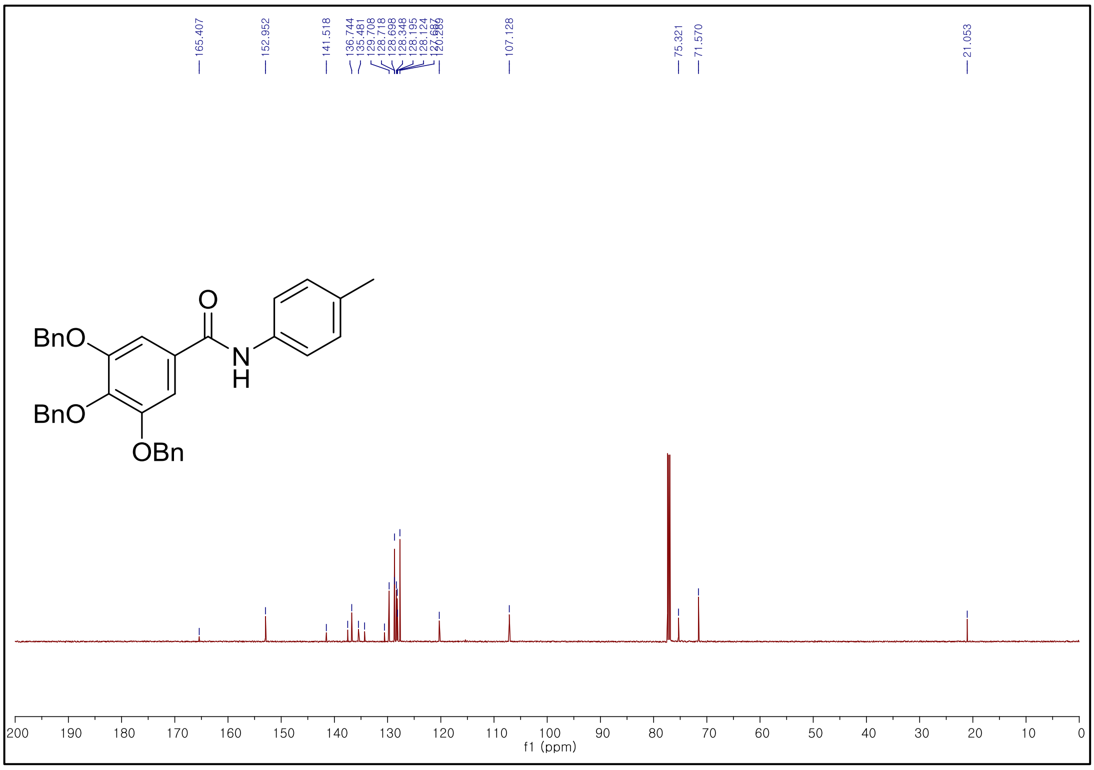

Figure. S2 ^{13}C-NMR (150 MHz, $CDCl_3$) of **3a**

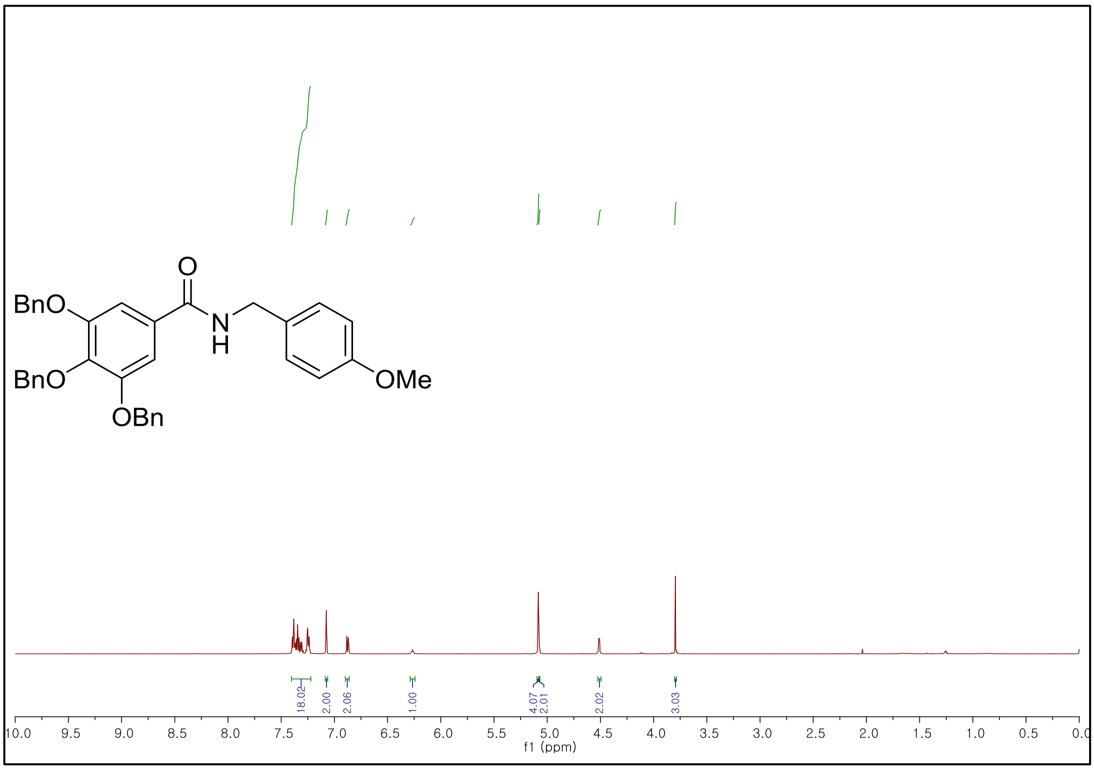

Figure S3. ^{1}H-NMR (600 MHz, $CDCl_3$) of **3b**

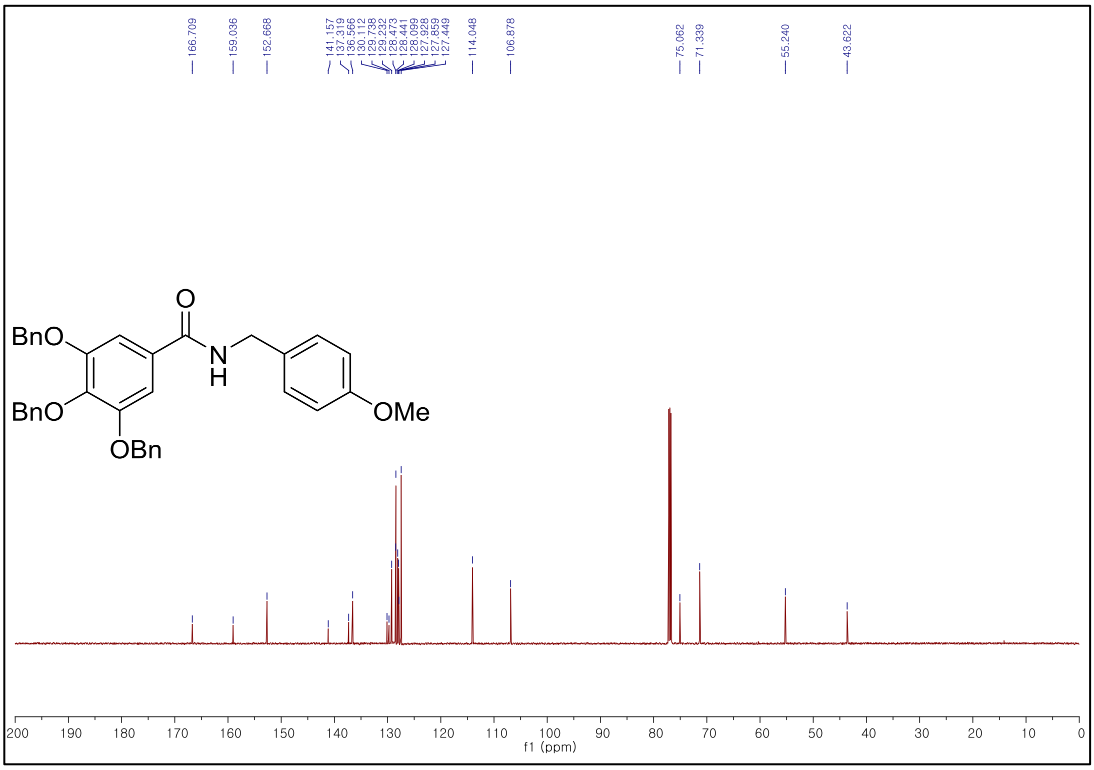

Figure S4. ^{13}C-NMR (150 MHz, $CDCl_3$) of **3b**.

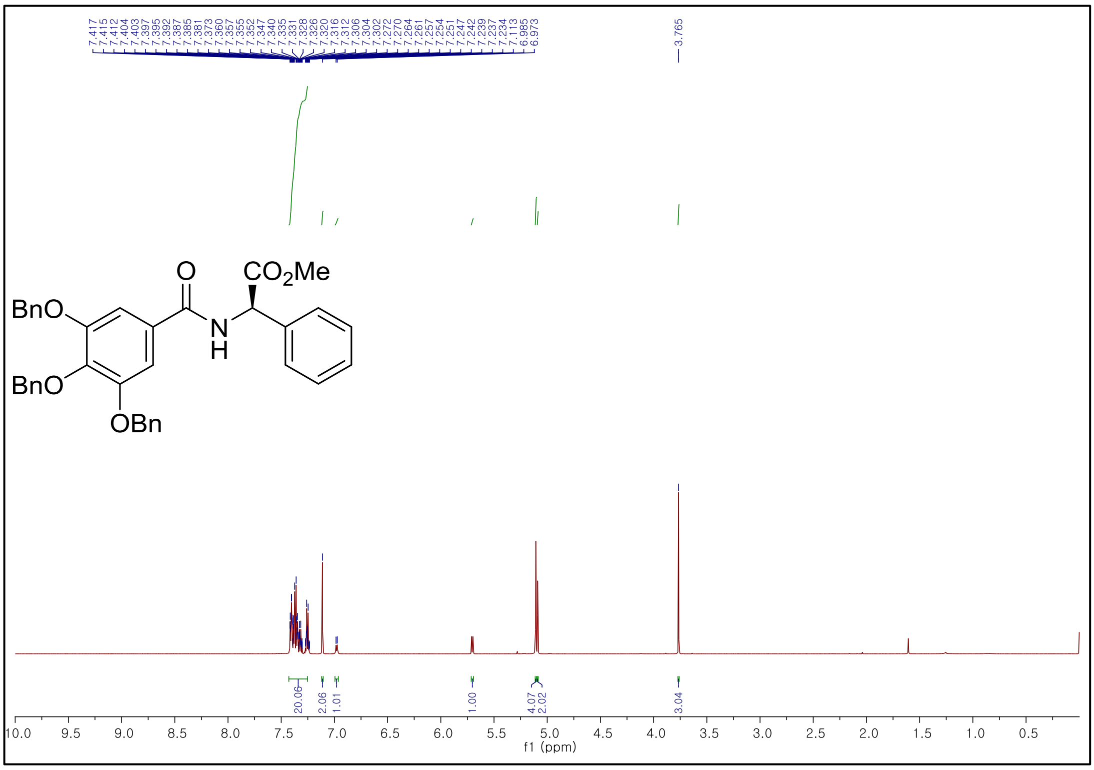

Figure S5. ^{1}H-NMR (600 MHz, $CDCl_3$) of **3c.**

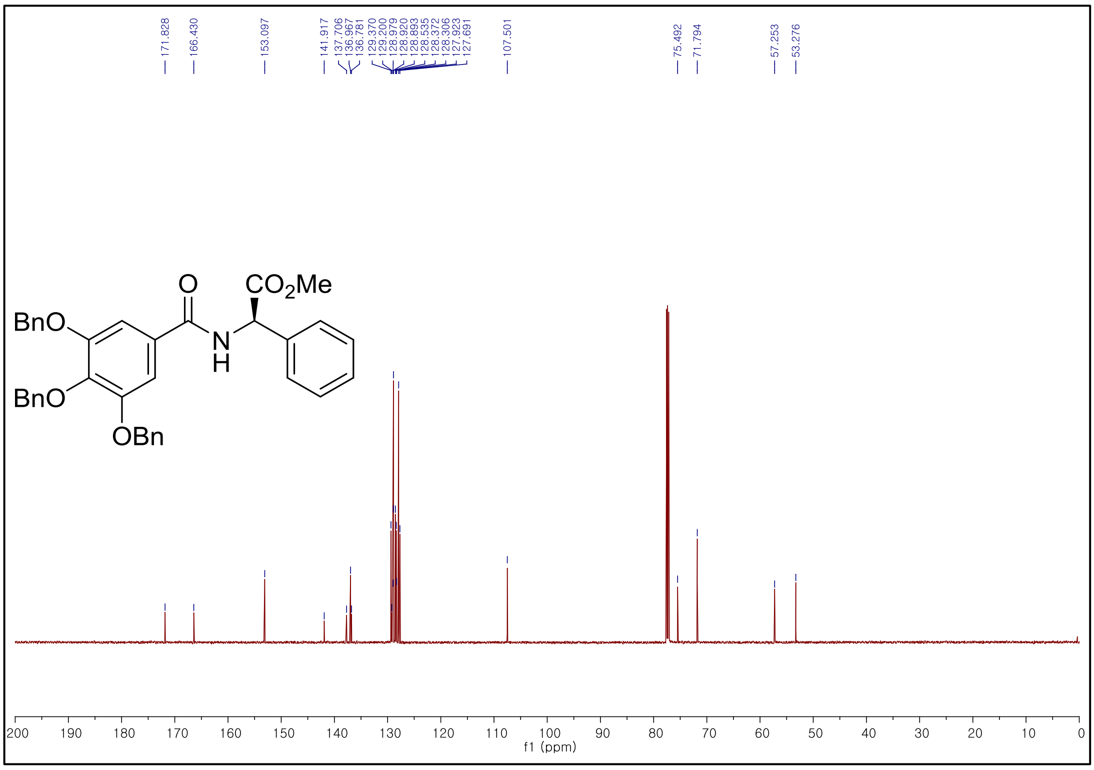

Figure S6. ^{13}C-NMR (150 MHz, $CDCl_3$) of **3c.**

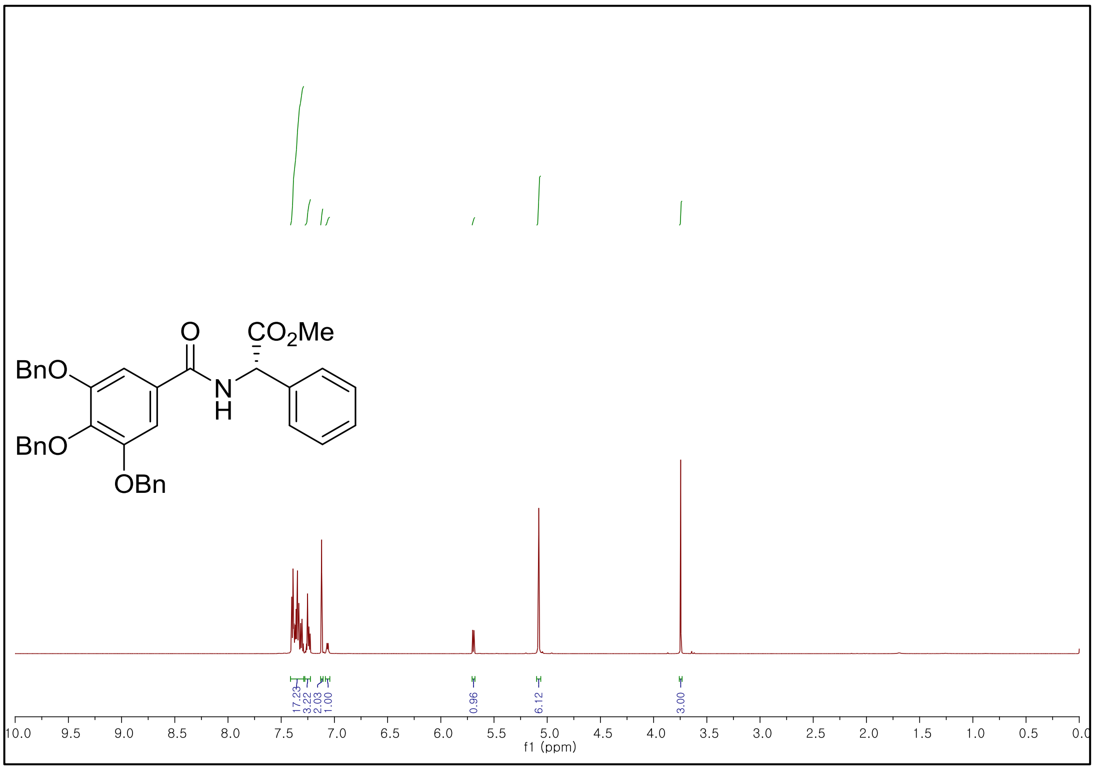

Figure S7. ^{1}H-NMR (600 MHz, $CDCl_3$) of **3d.**

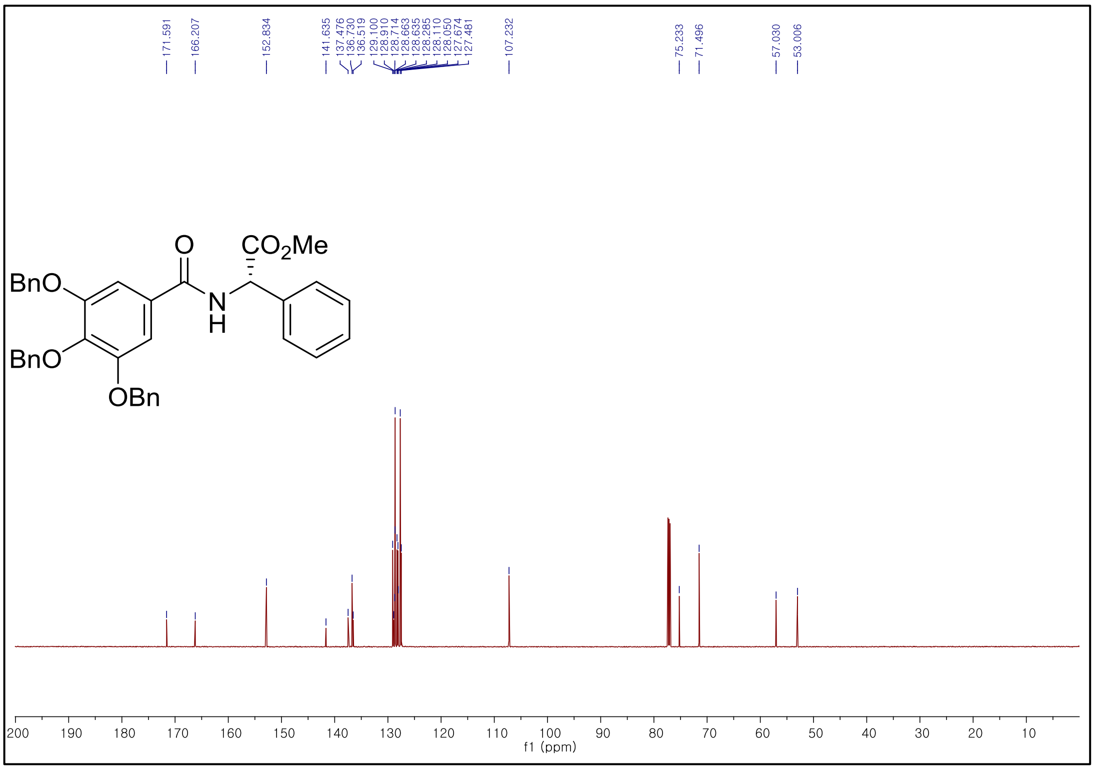

Figure S8. ^{13}C-NMR (150 MHz, $CDCl_3$) of **3d.**

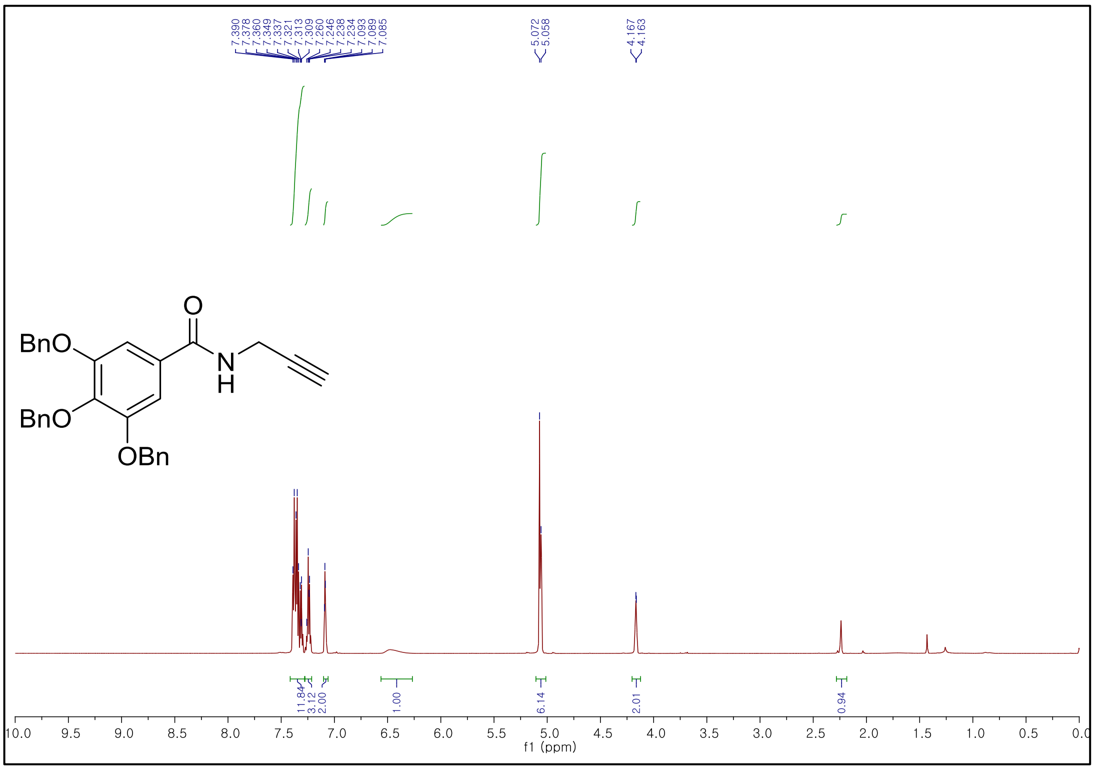

Figure S9. ^{1}H-NMR (600 MHz, $CDCl_3$) of **3e**

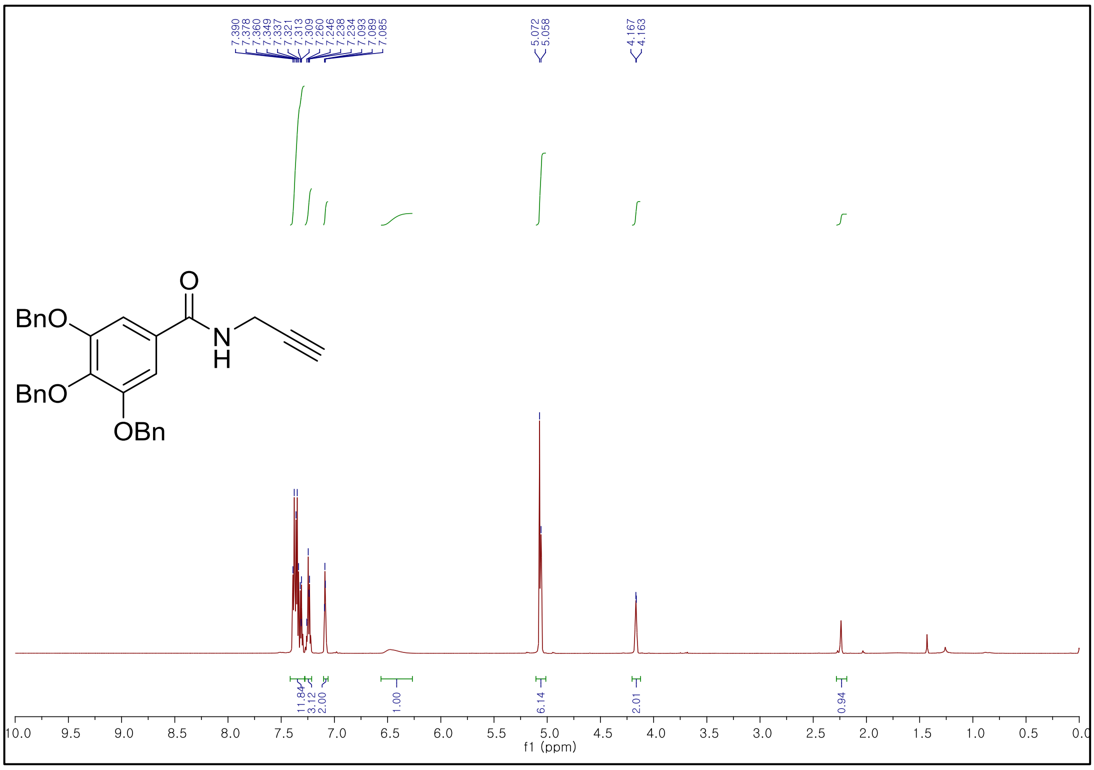

Figure S10. ^{13}C-NMR (150 MHz, $CDCl_3$) of **3e.**

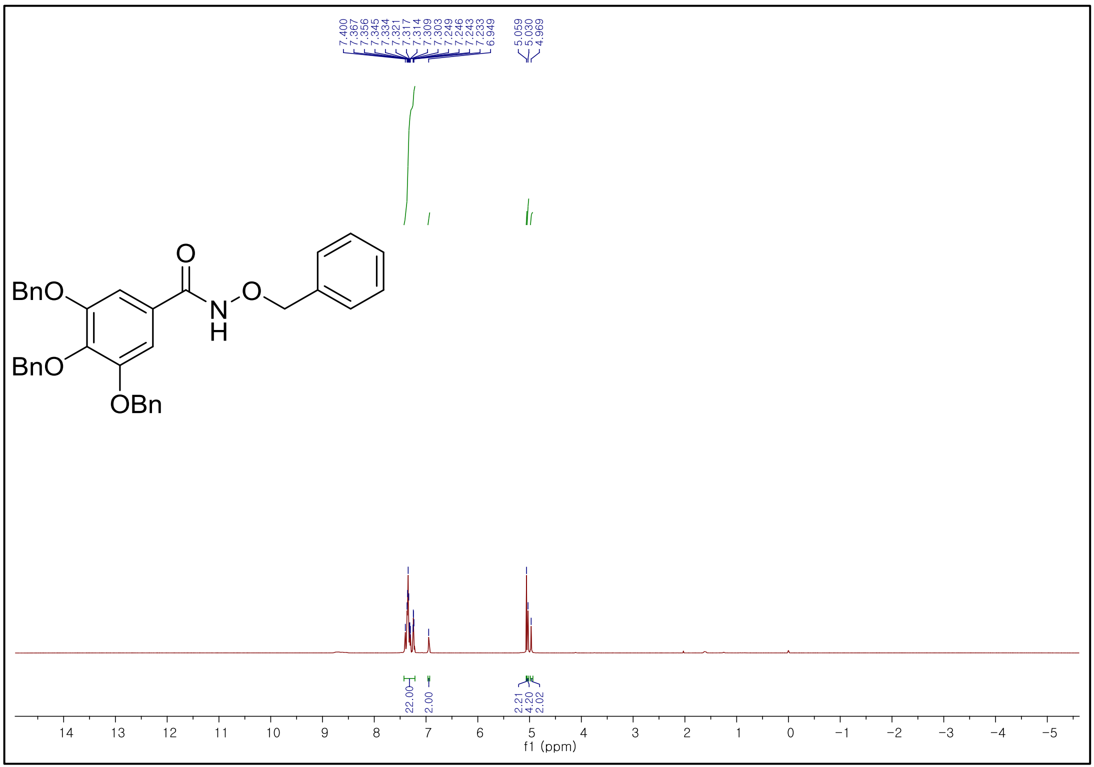

Figure S11. ^{1}H-NMR (600 MHz, $CDCl_3$) of **3f**.

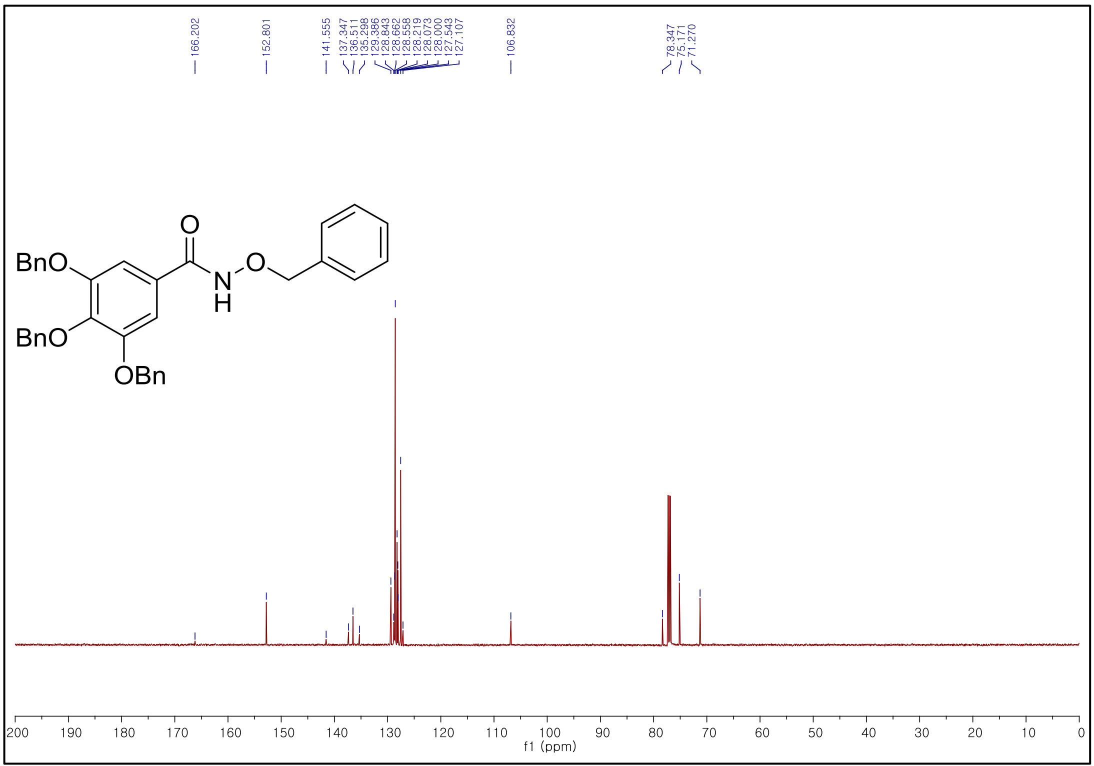

Figure S12. ^{13}C-NMR (150 MHz, $CDCl_3$) of **3f**.

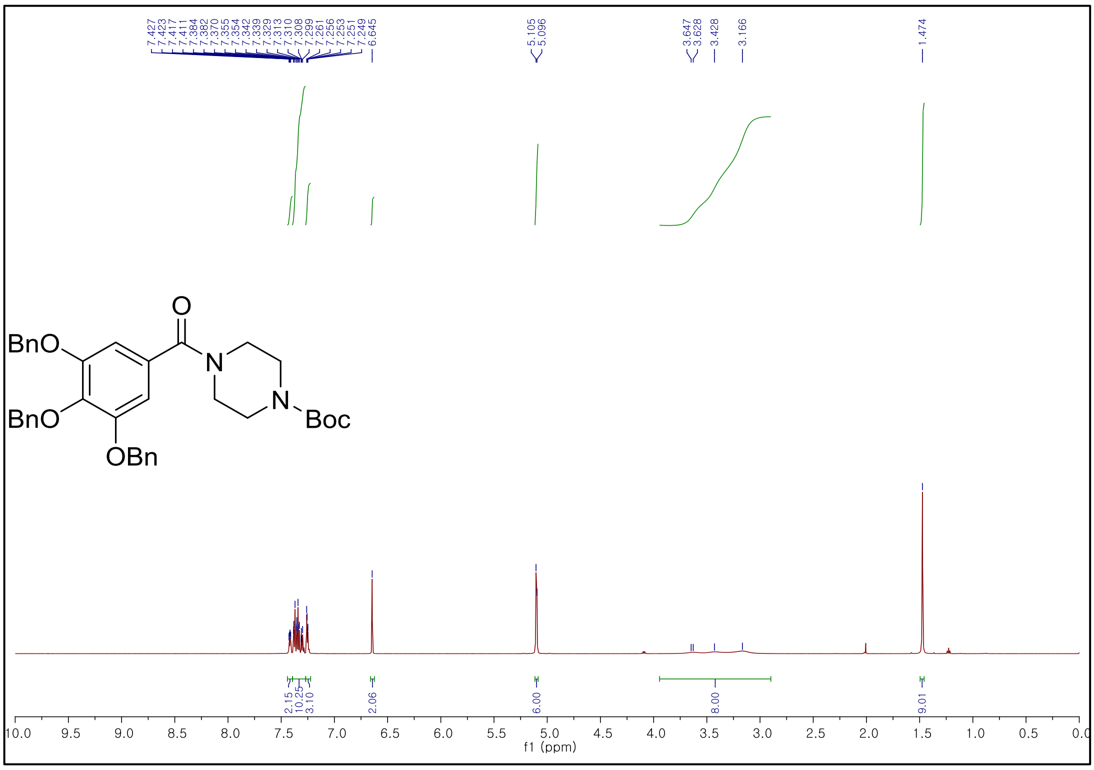

Figure S13. ^{1}H-NMR (600 MHz, $CDCl_3$) of **3g**.

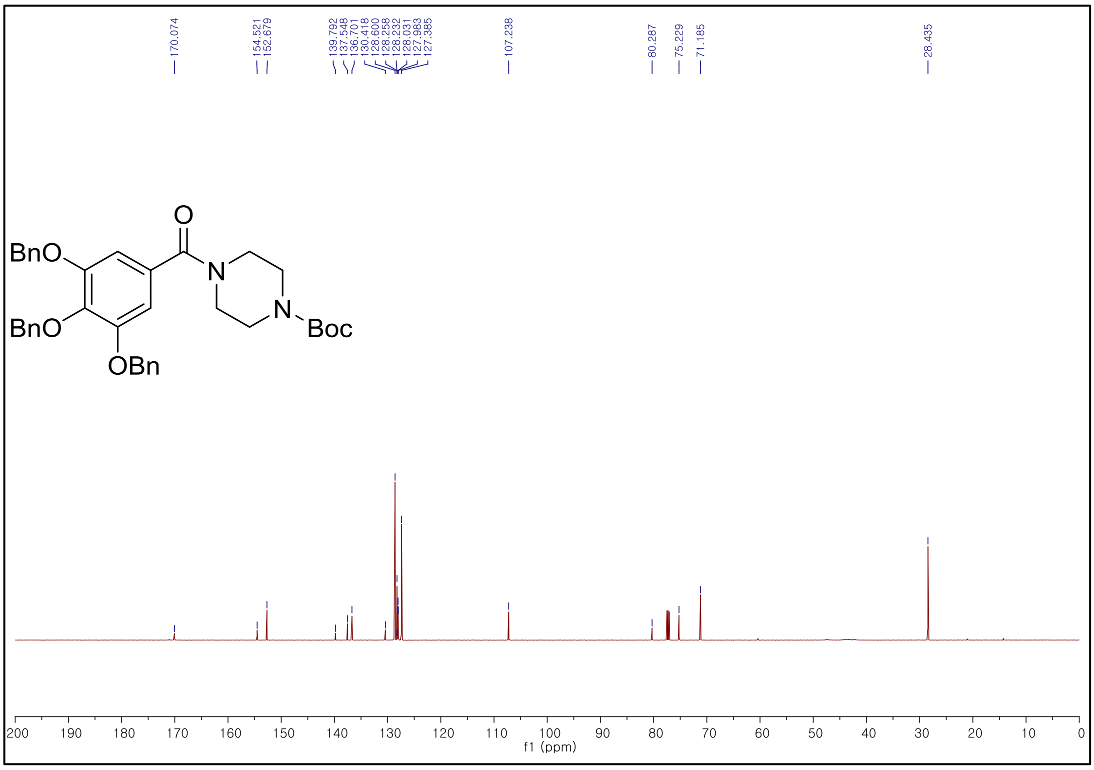

Figure S14. ^{13}C-NMR (150 MHz, $CDCl_3$) of **3g**.

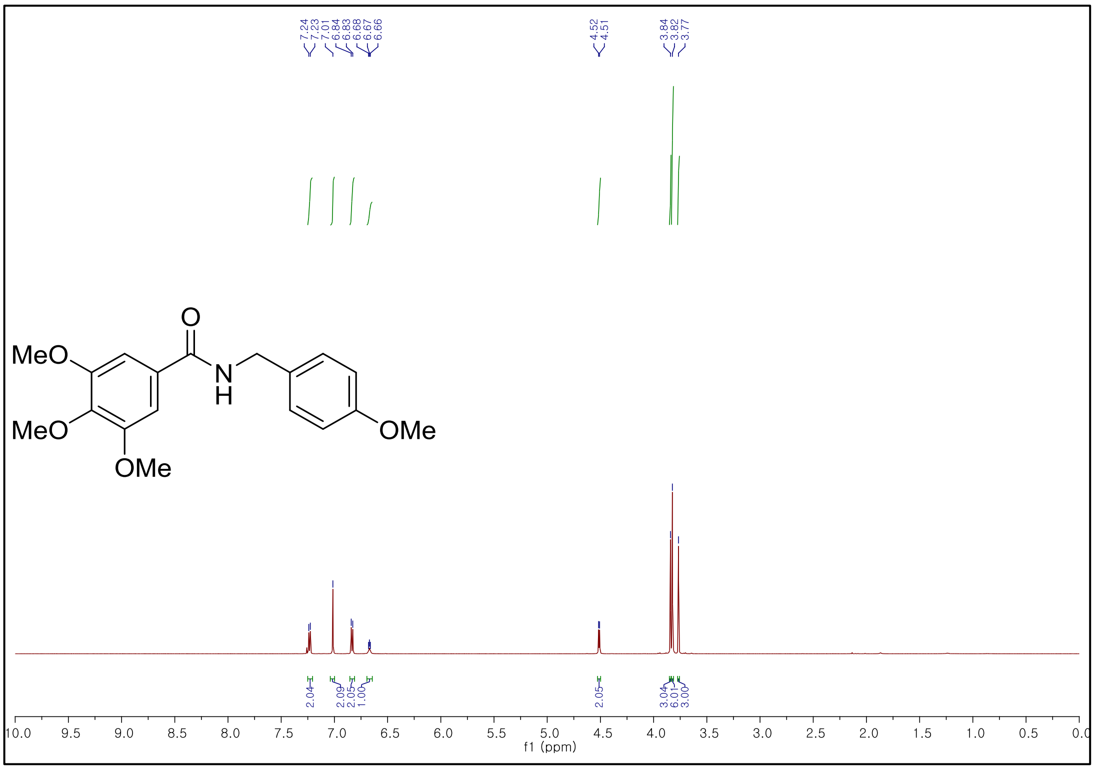

Figure S15. ^{1}H-NMR (600 MHz, $CDCl_3$) of **4b**.

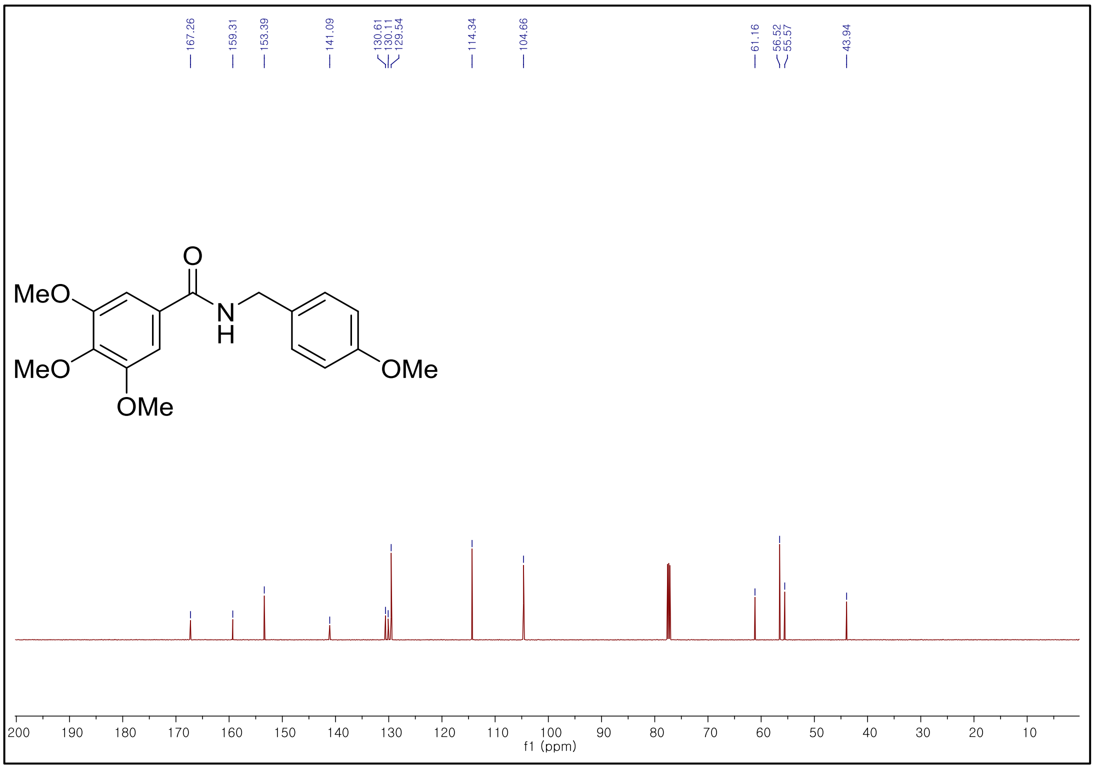

Figure S16. ^{13}C-NMR (150 MHz, $CDCl_3$) of **4b**.

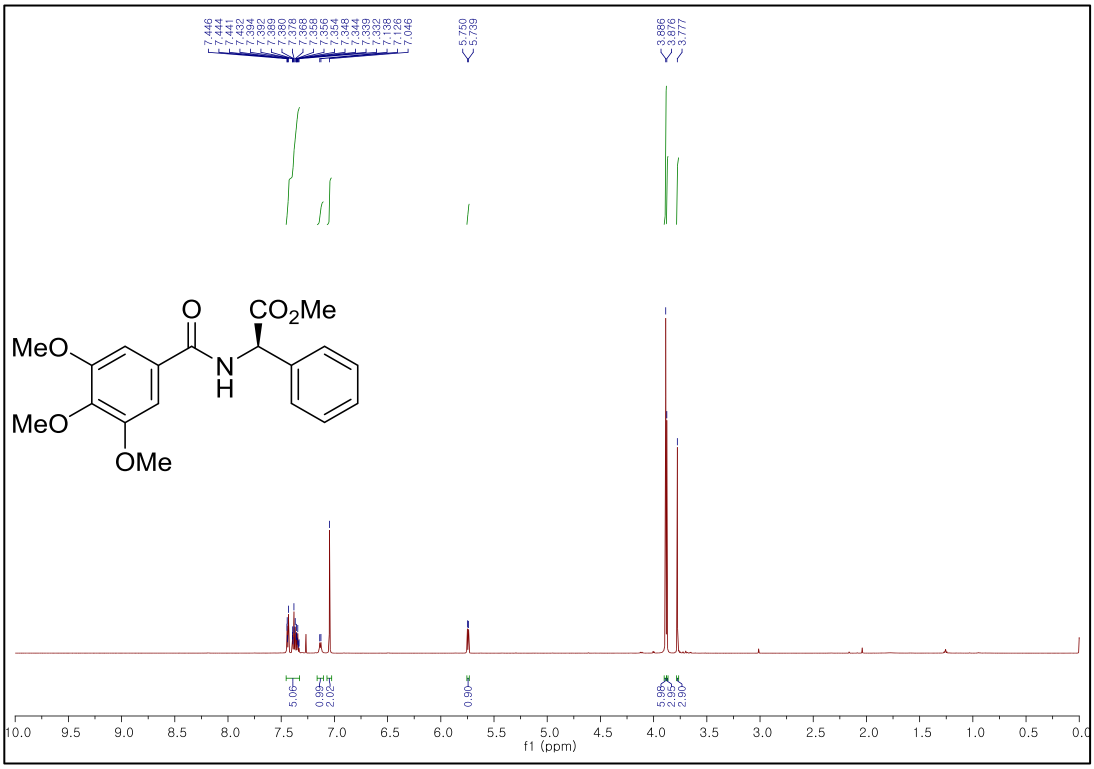

Figure S17. ^{1}H-NMR (600 MHz, $CDCl_3$) of **4c**.

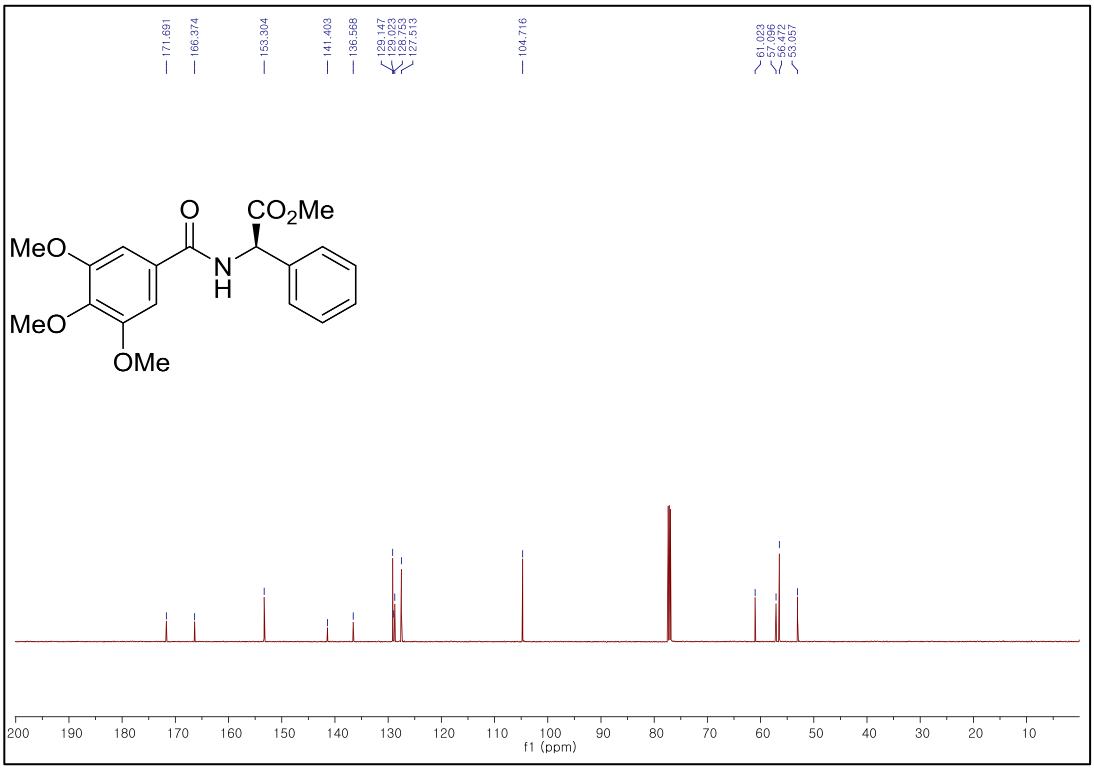

Figure S18. ^{13}C-NMR (150 MHz, $CDCl_3$) of **4c**.

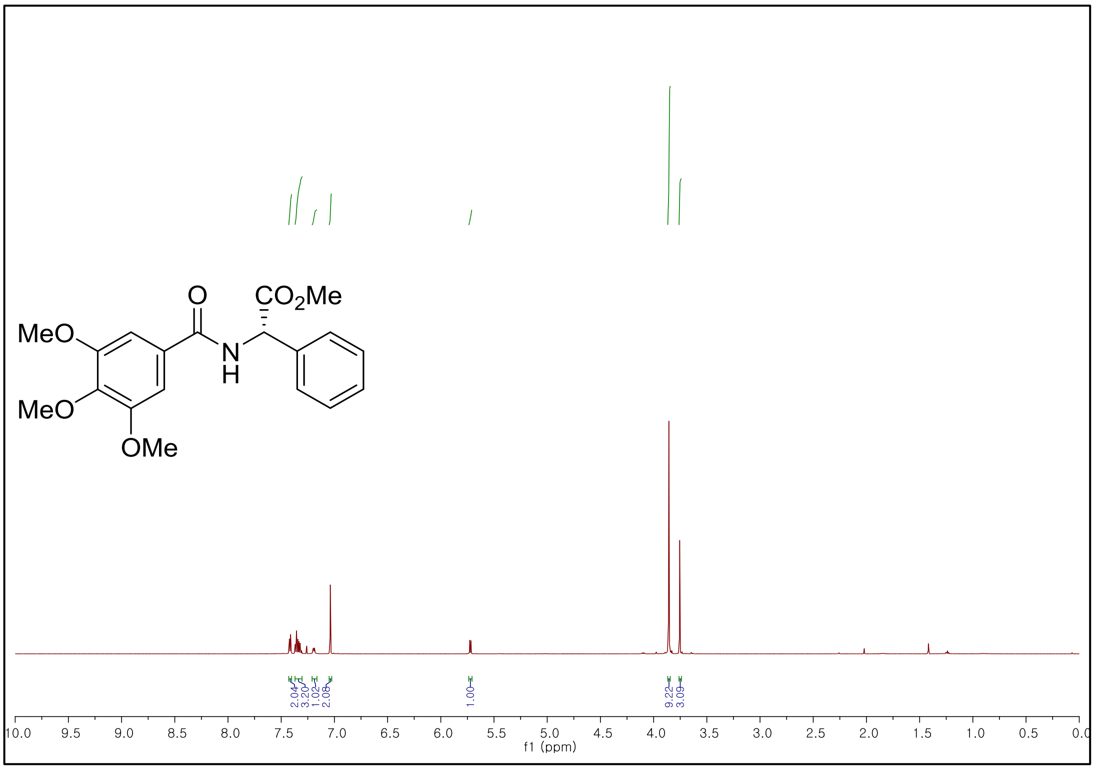

Figure S19. ^{1}H-NMR (600 MHz, $CDCl_3$) of **4d**.

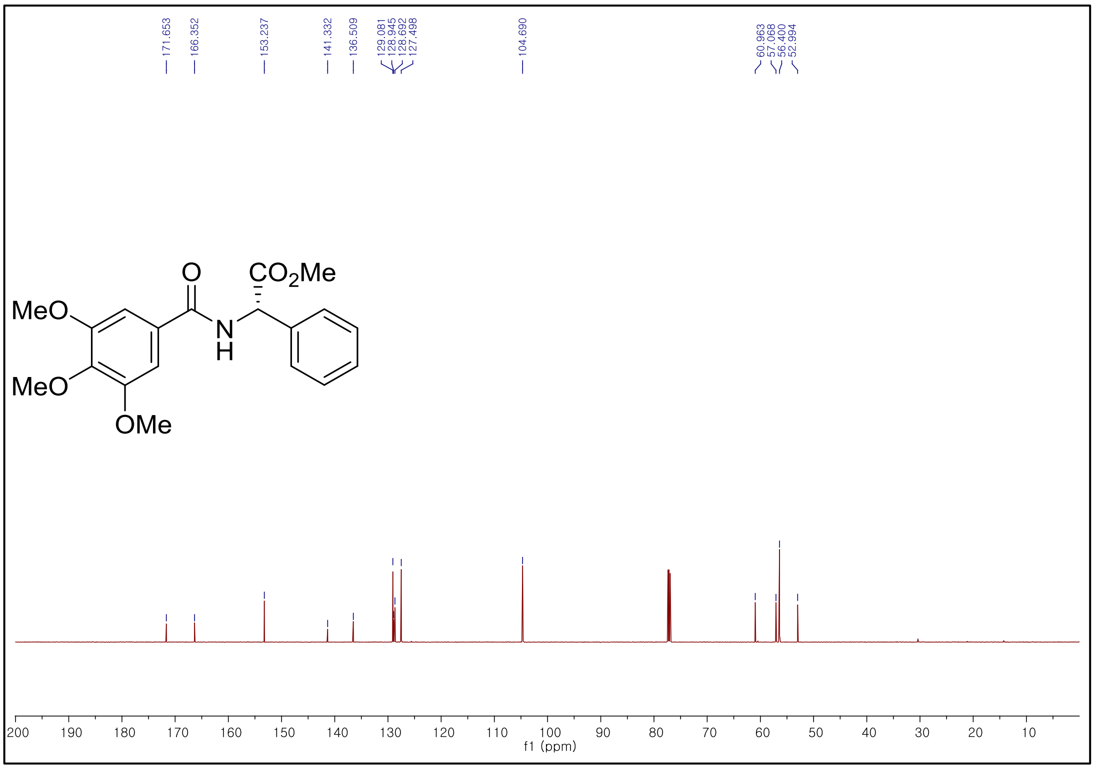

Figure S20. ^{13}C-NMR (150 MHz, $CDCl_3$) of **4d**.

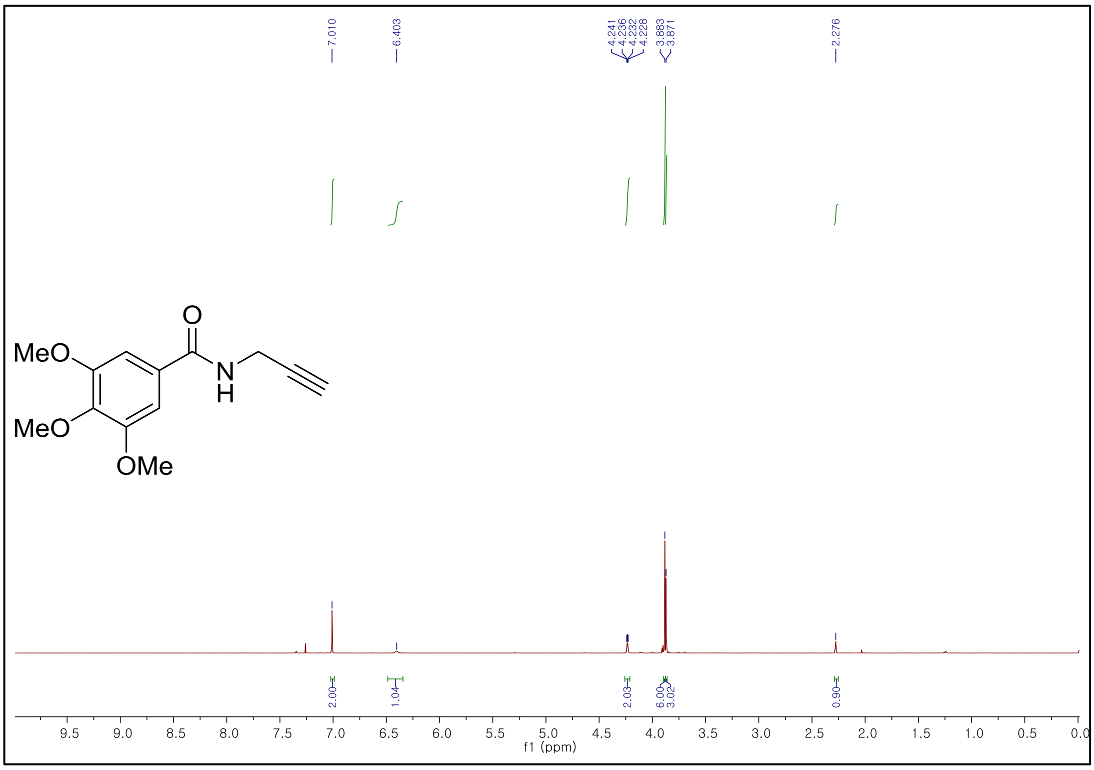

Figure S21. ^{1}H-NMR (600 MHz, $CDCl_3$) of **4e**.

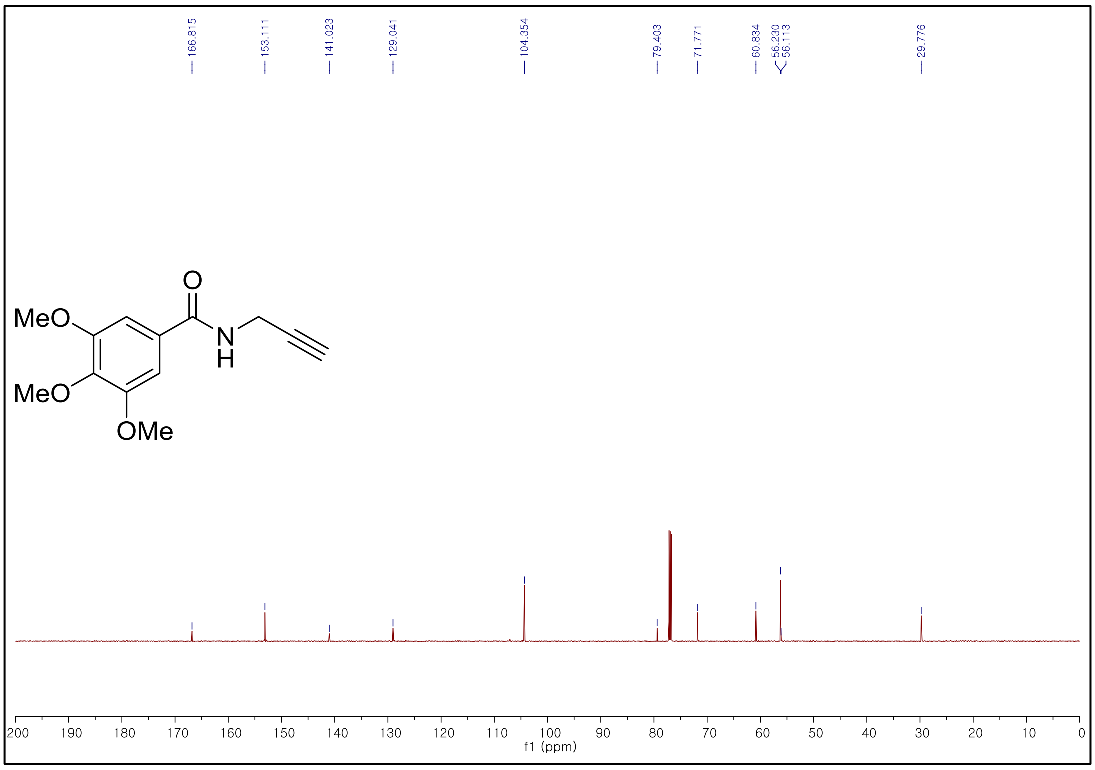

Figure S22. ^{13}C-NMR (150 MHz, $CDCl_3$) of **4e**.

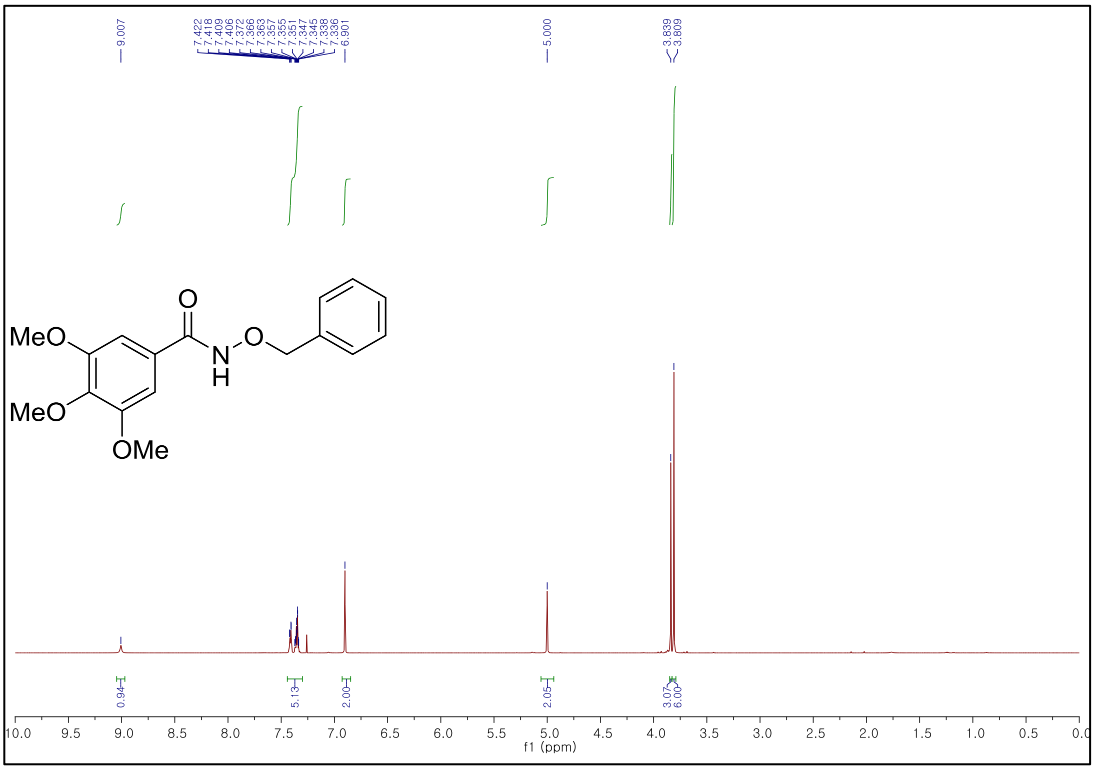

Figure S23. ^{1}H-NMR (600 MHz, $CDCl_3$) of **4f**.

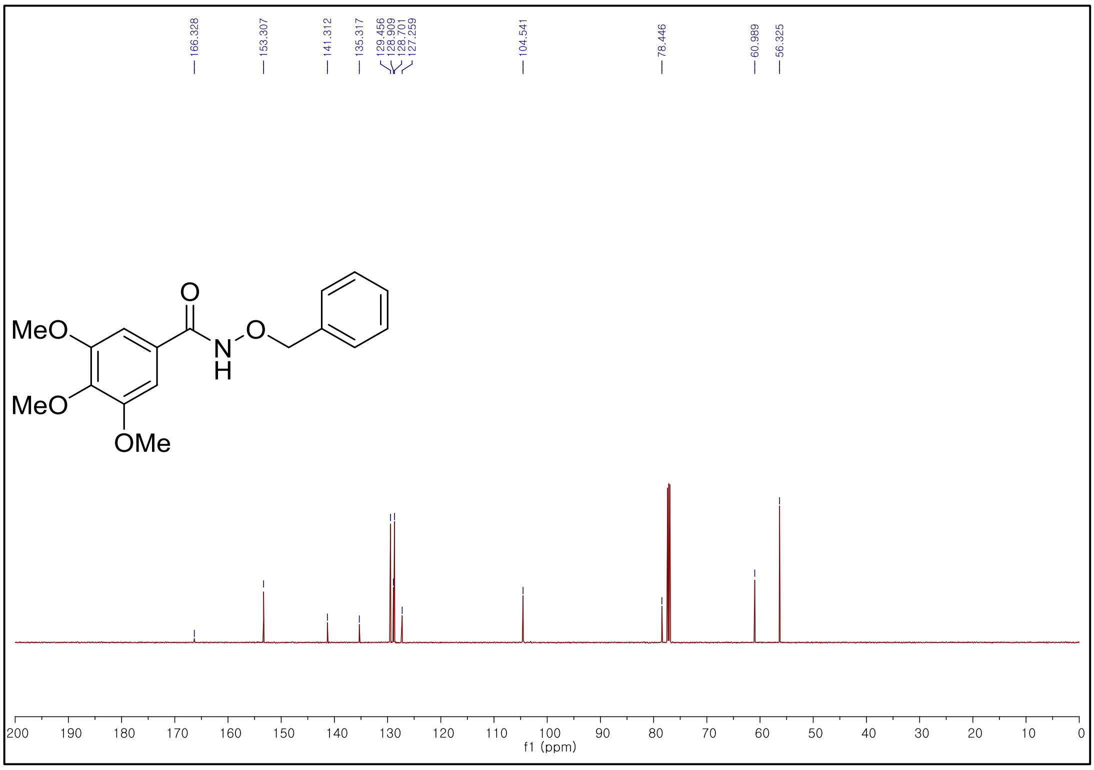

Figure S24. ^{13}C-NMR (150 MHz, $CDCl_3$) of **4f**.

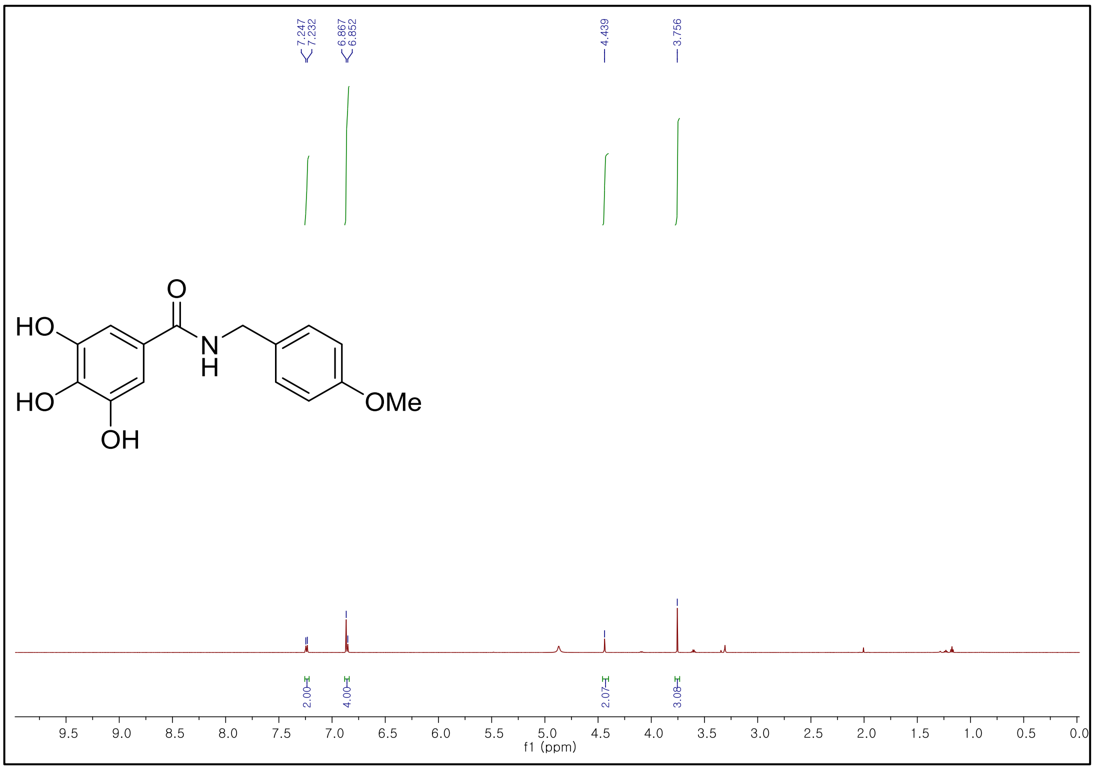

Figure S25. ^{1}H-NMR (600 MHz, Methanol-d4) of **5b**.

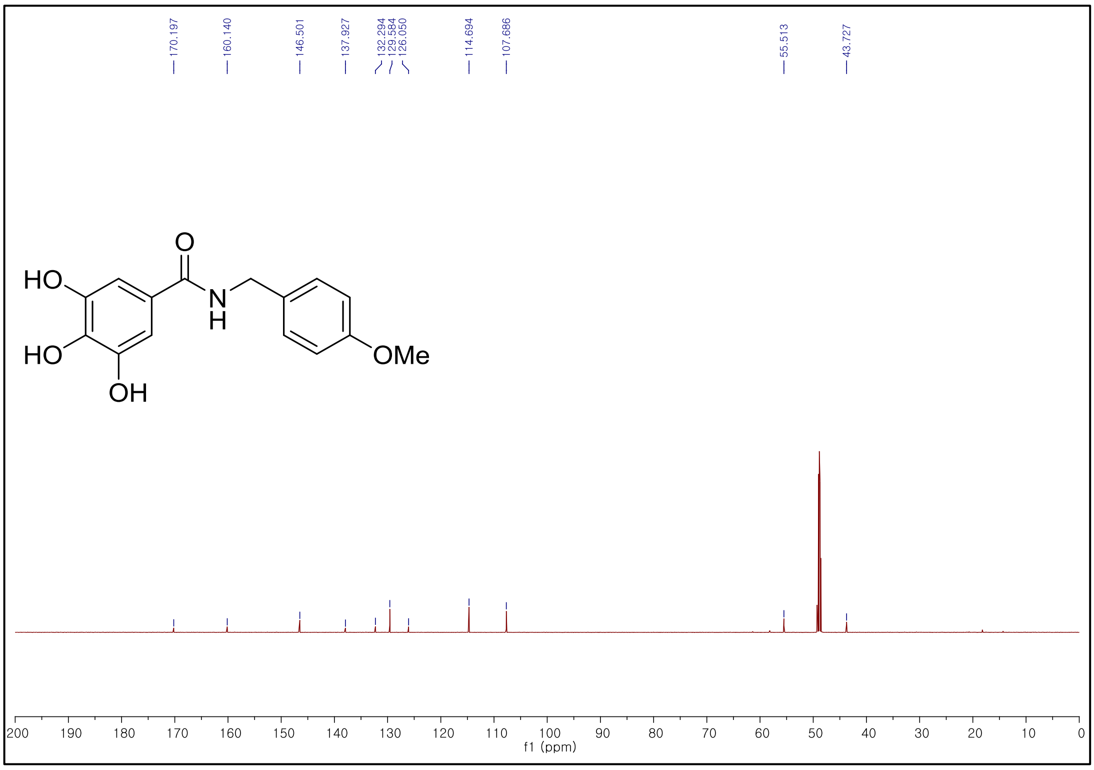

Figure S26. ^{13}C-NMR (150 MHz, Methanol-d4) of **5b**.

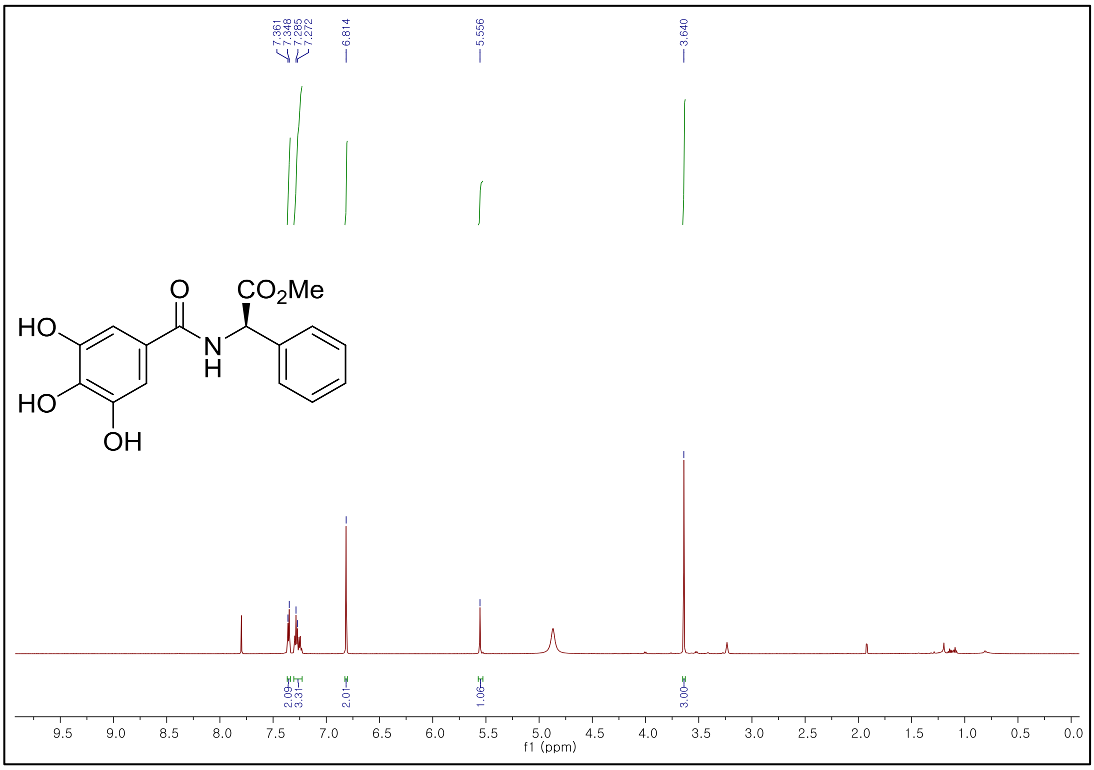

Figure S27. ^{1}H-NMR (600 MHz, Methanol-d4) of **5c**.

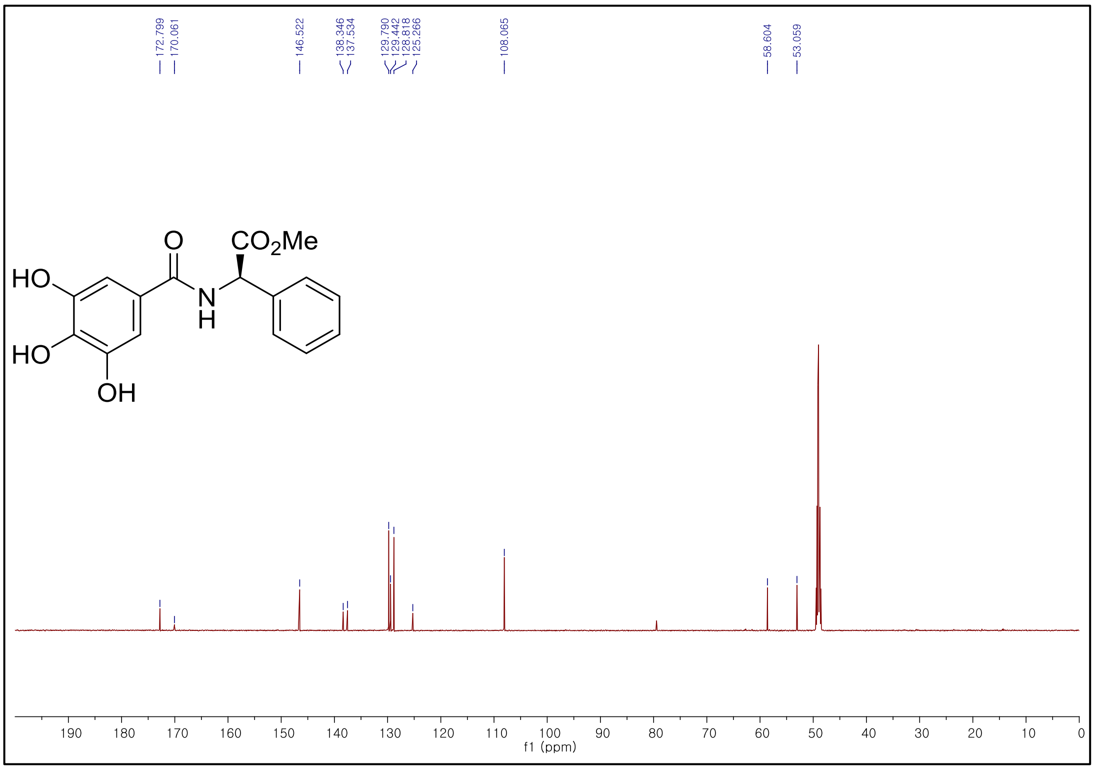

Figure S28. ^{13}C-NMR (150 MHz, Methanol-d4) of **5c**.

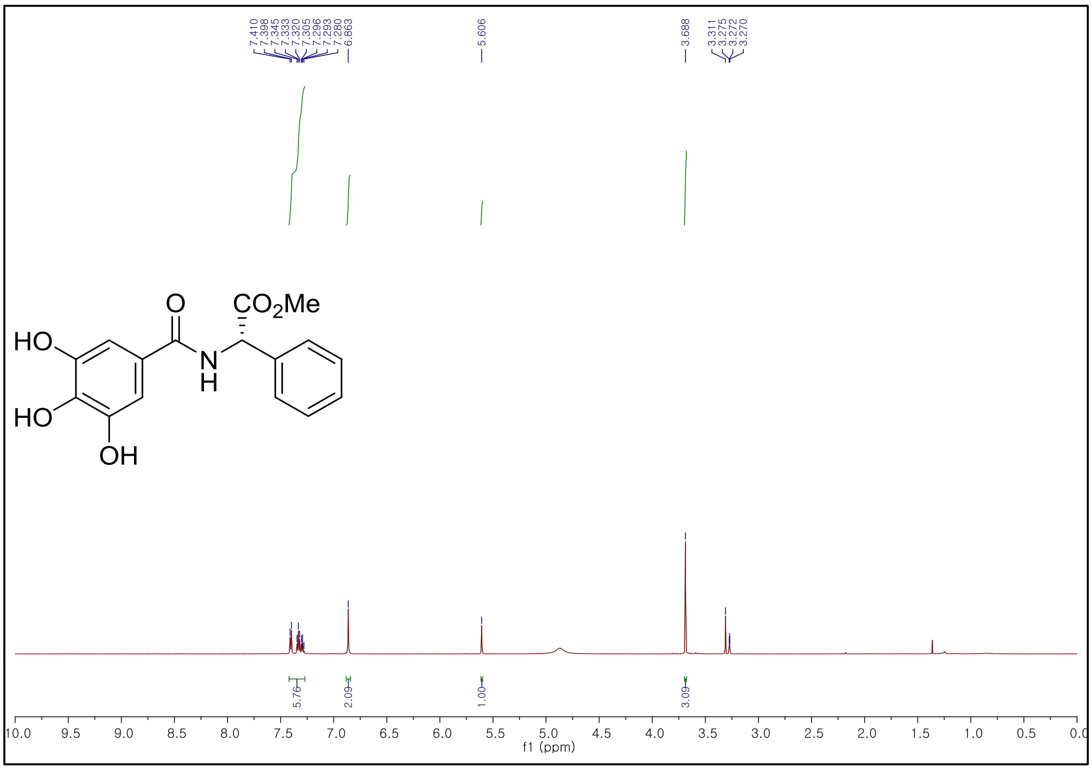

Figure S29. ^{1}H-NMR (600 MHz, Methanol-d4) of **5d**.

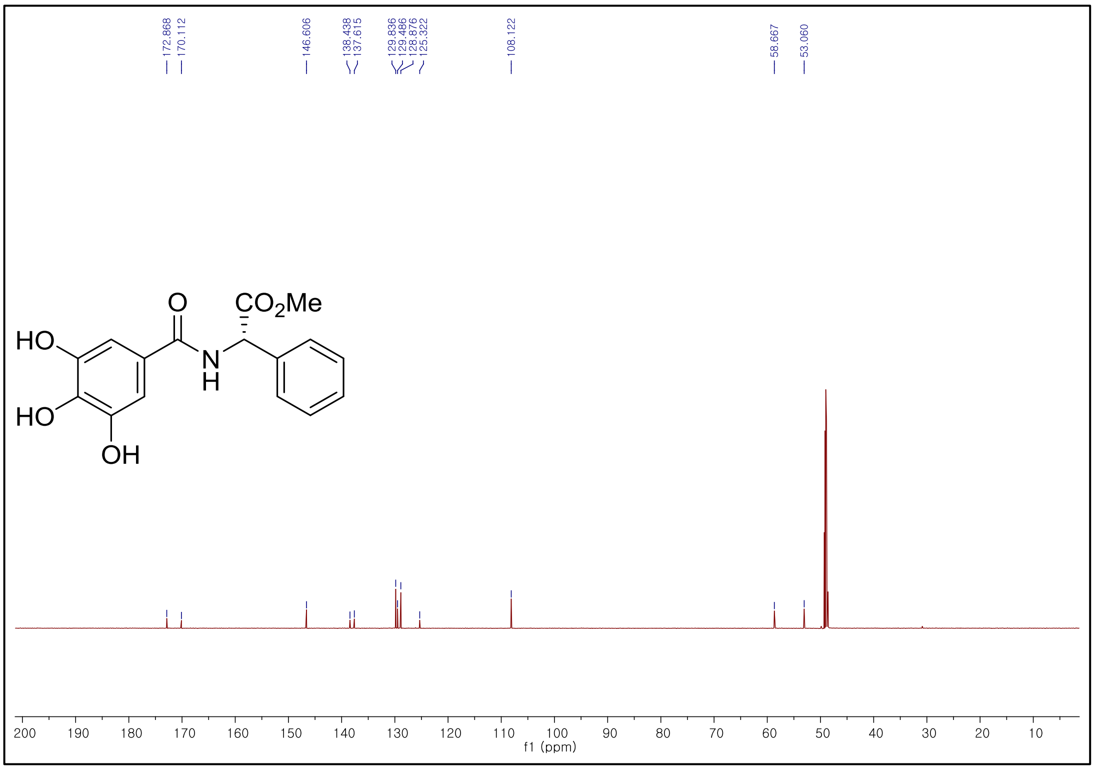

Figure S30. ^{13}C-NMR (150 MHz, Methanol-d4) of **5d**.

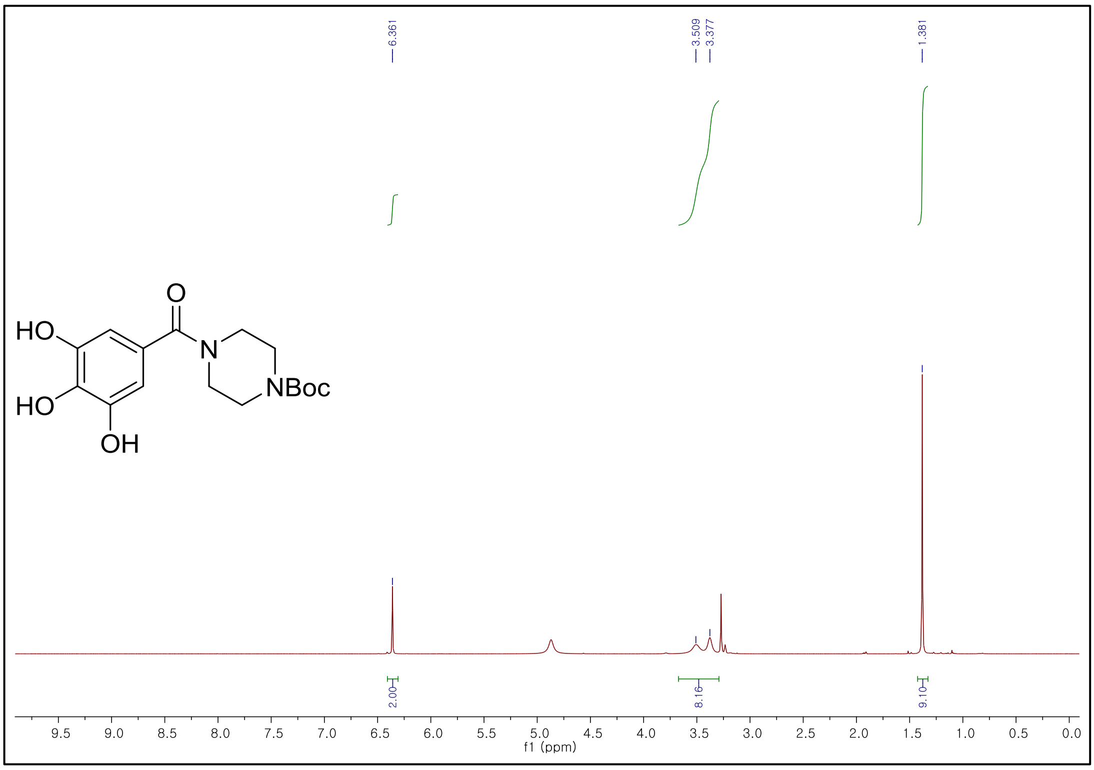

Figure S31. ^{1}H-NMR (600 MHz, Methanol-d4) of **5g.**

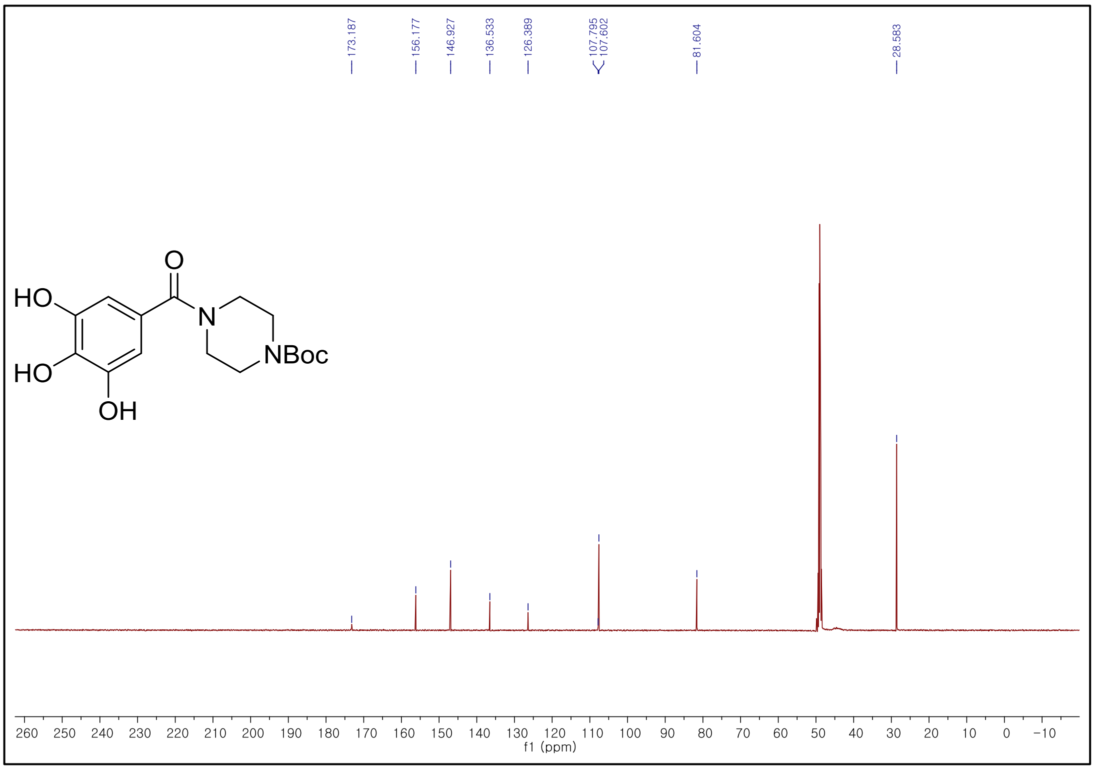

Figure S32. ^{13}C-NMR (150 MHz, Methanol-d4) of **5g**.

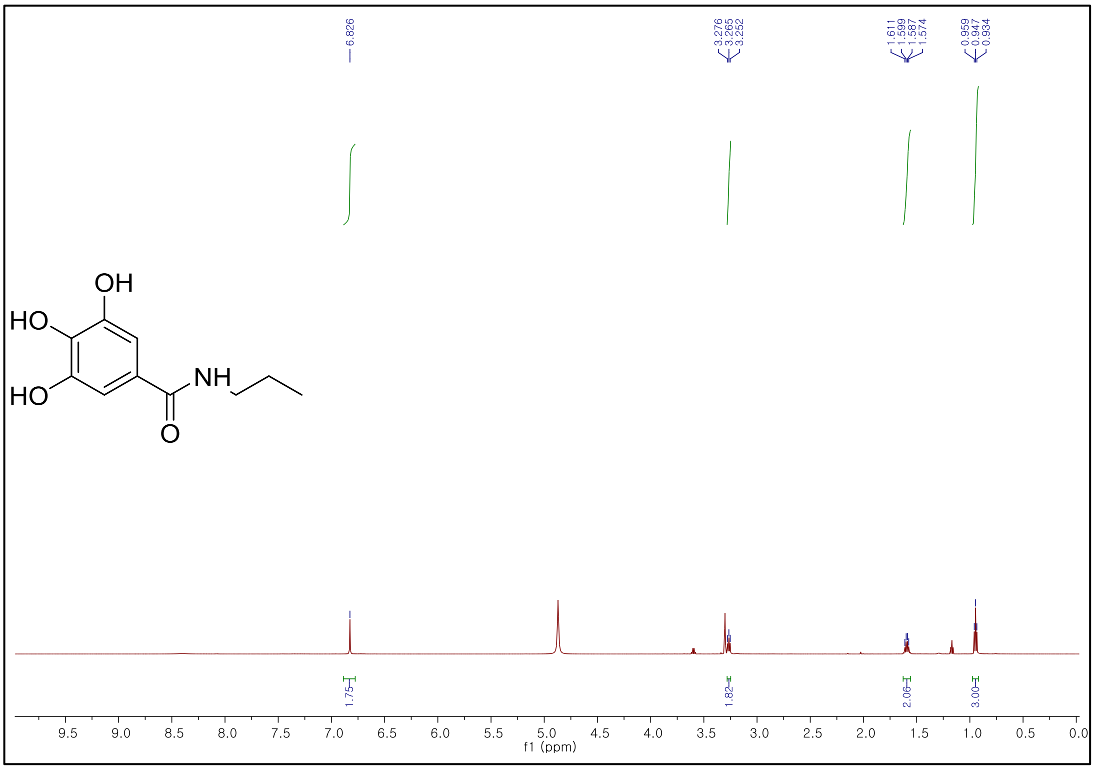

Figure S33. ^{1}H-NMR (600 MHz, Methanol-d4) of **5h**.

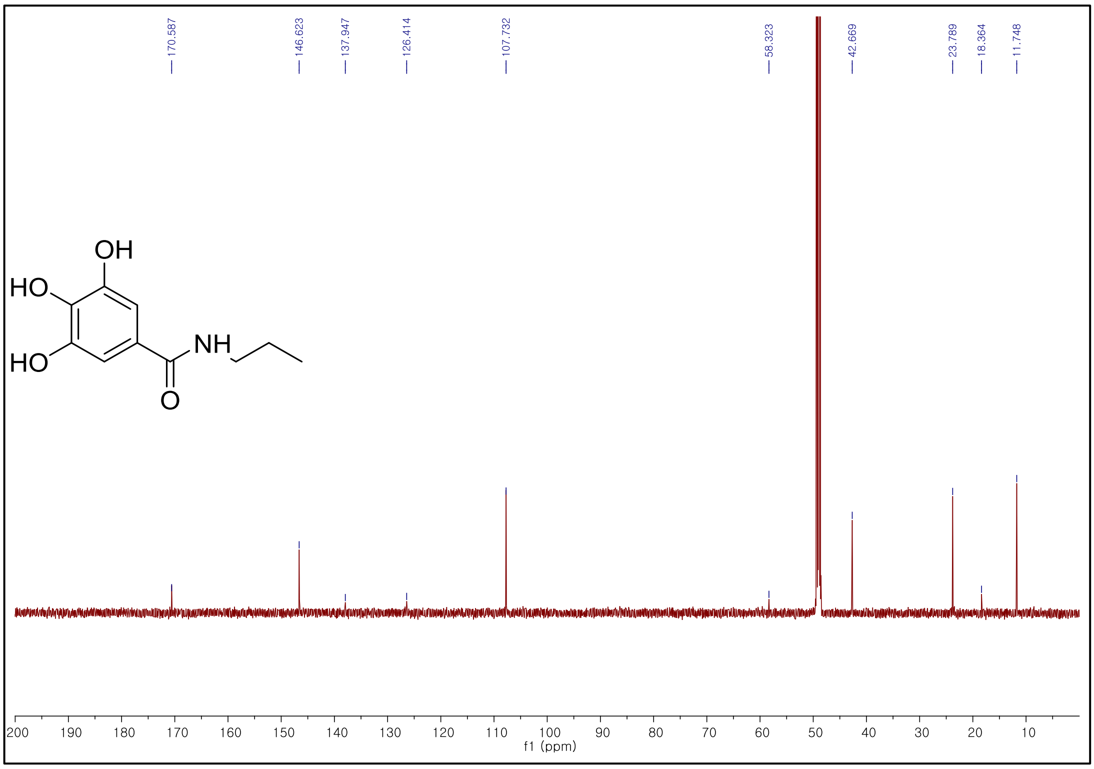

Figure S34. ^{13}C-NMR (150 MHz, Methanol-d4) of **5h**.

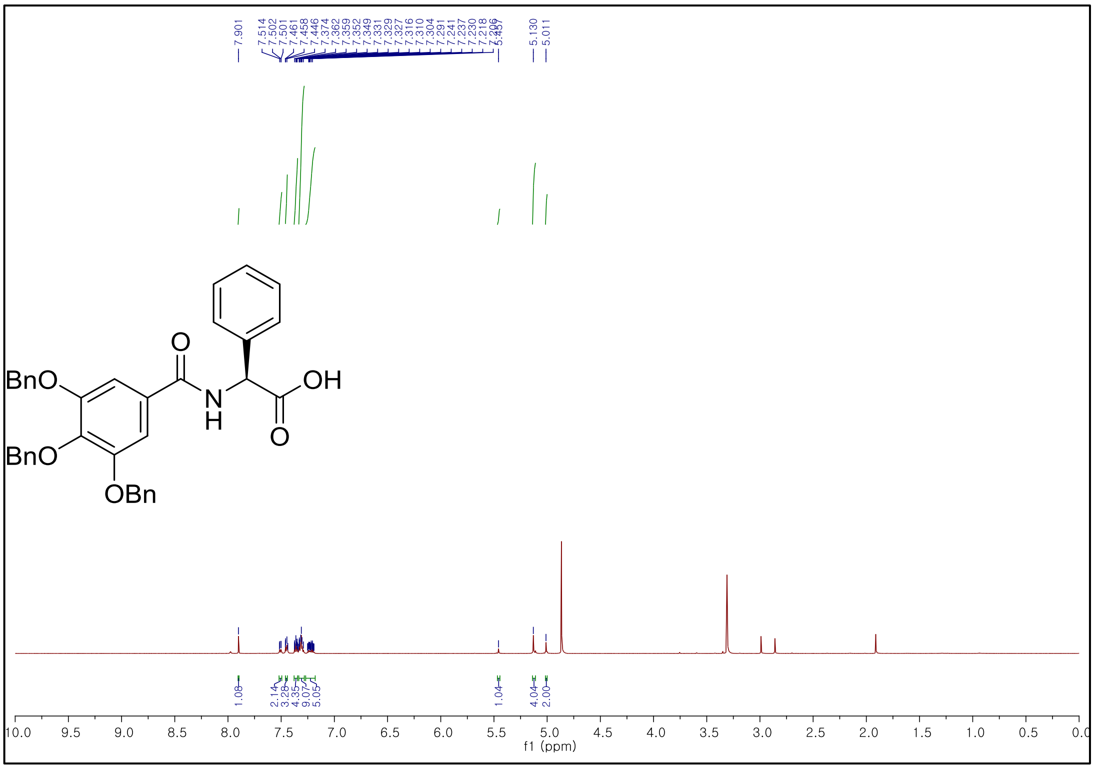

Figure S35. ^{1}H-NMR (600 MHz, Methanol-d4) of **6a**.

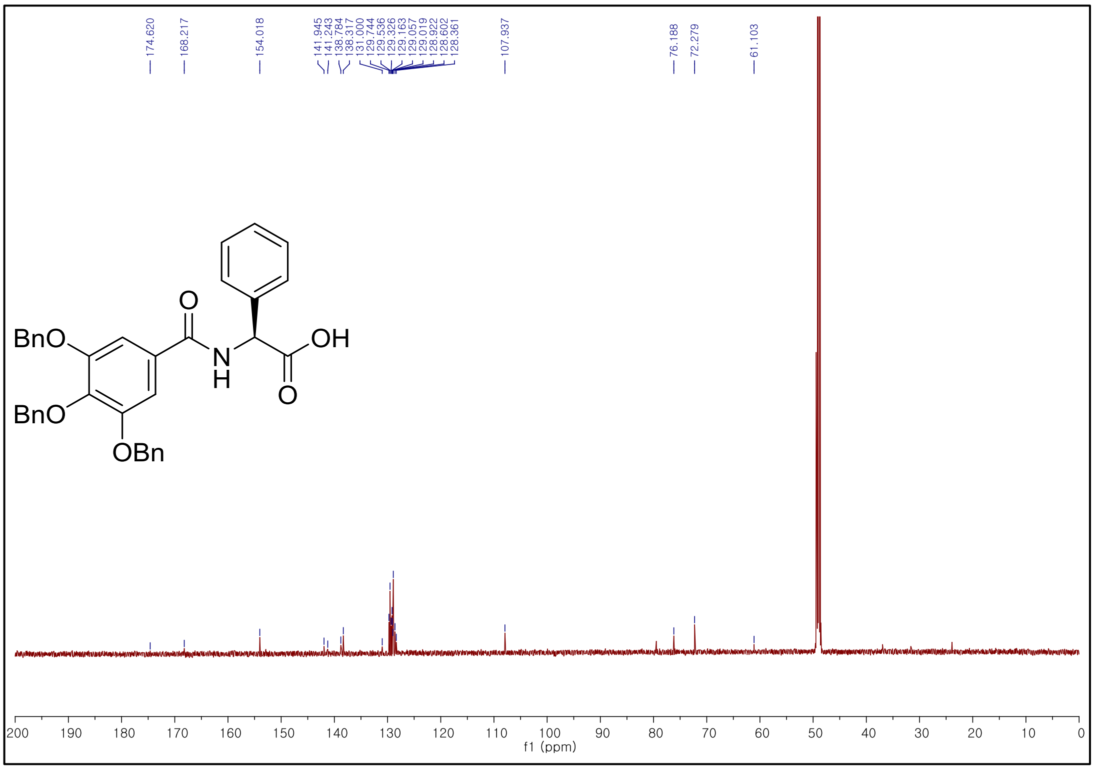

Figure S36. ^{13}C-NMR (150 MHz, Methanol-d4) of **6a**.

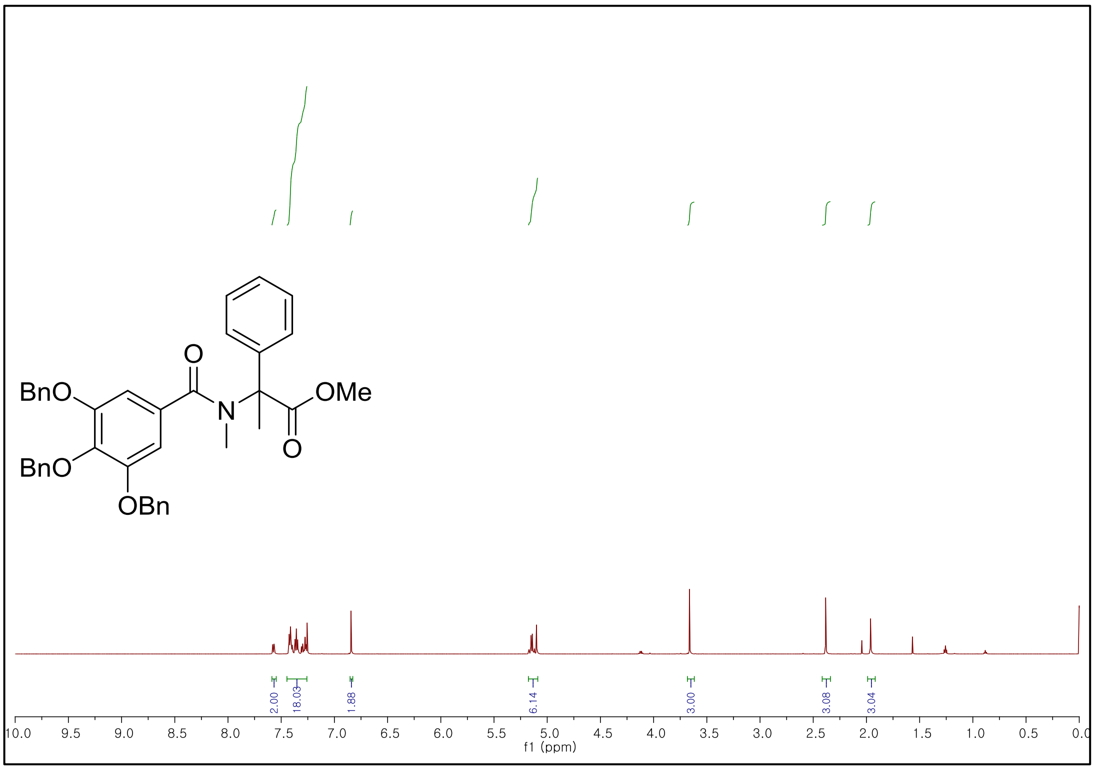

Figure S37. ^{1}H-NMR (600 MHz, $CDCl_3$) of **6b**.

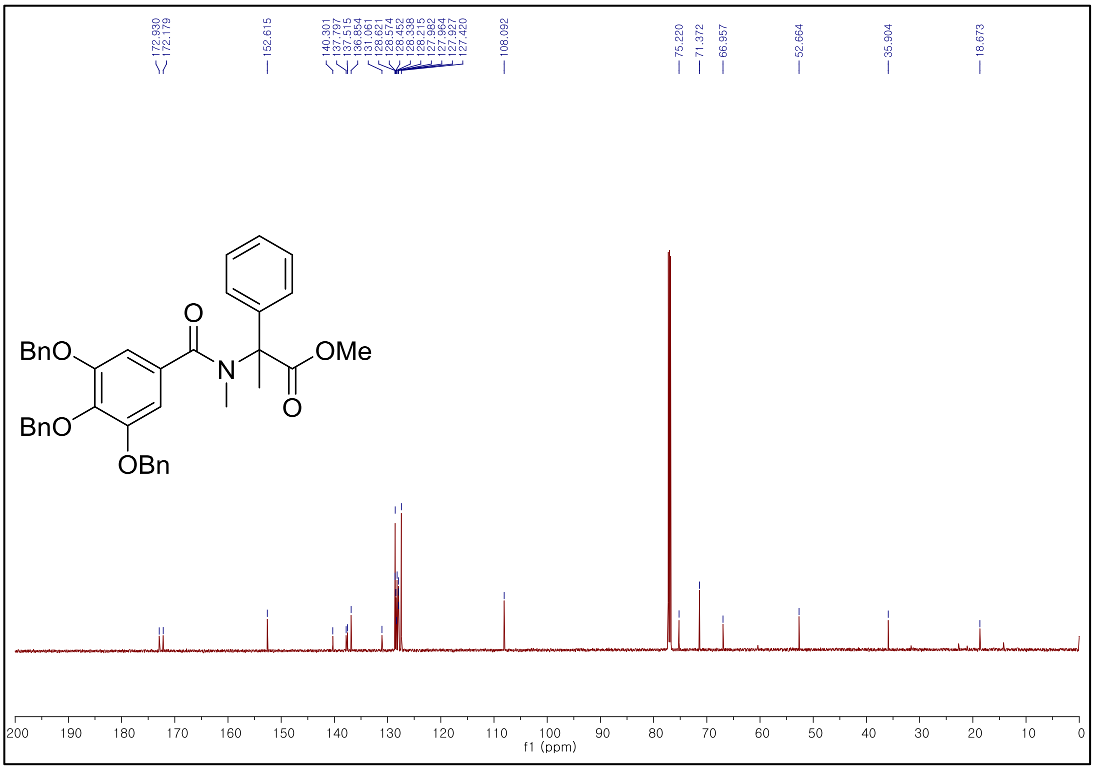

Figure S38. ^{13}C-NMR (150 MHz, $CDCl_3$) of **6b**.

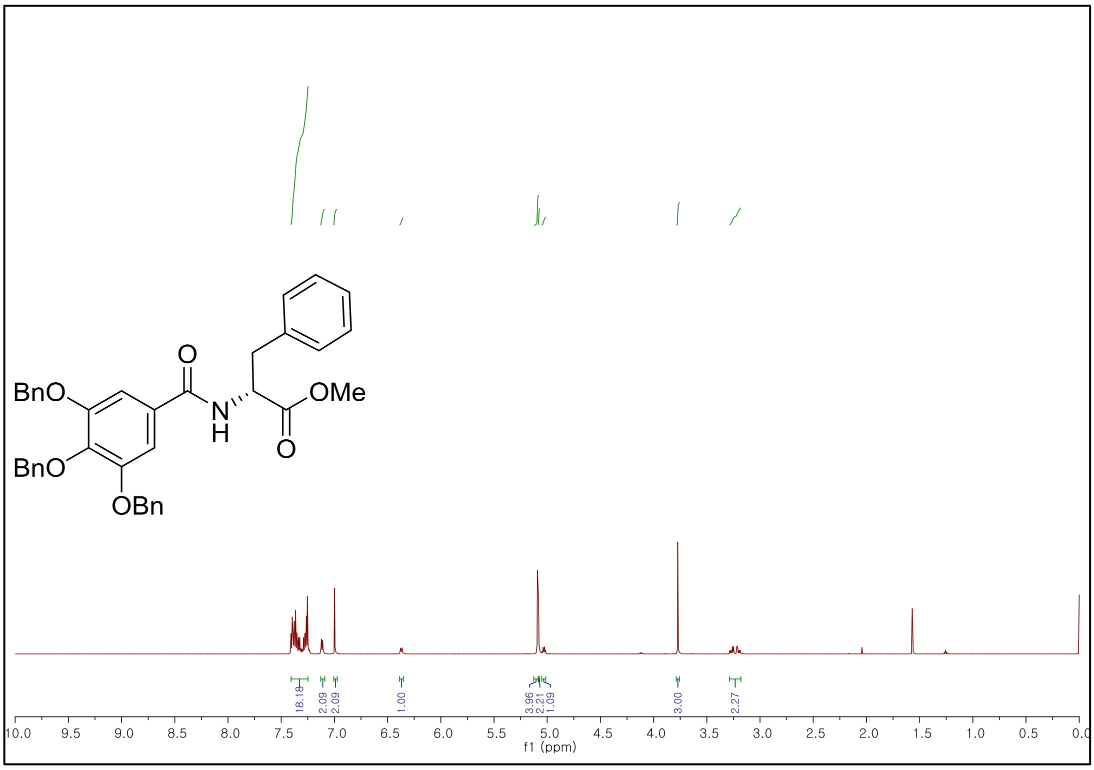

Figure S39. ^{1}H-NMR (600 MHz, $CDCl_3$) of **6c**.

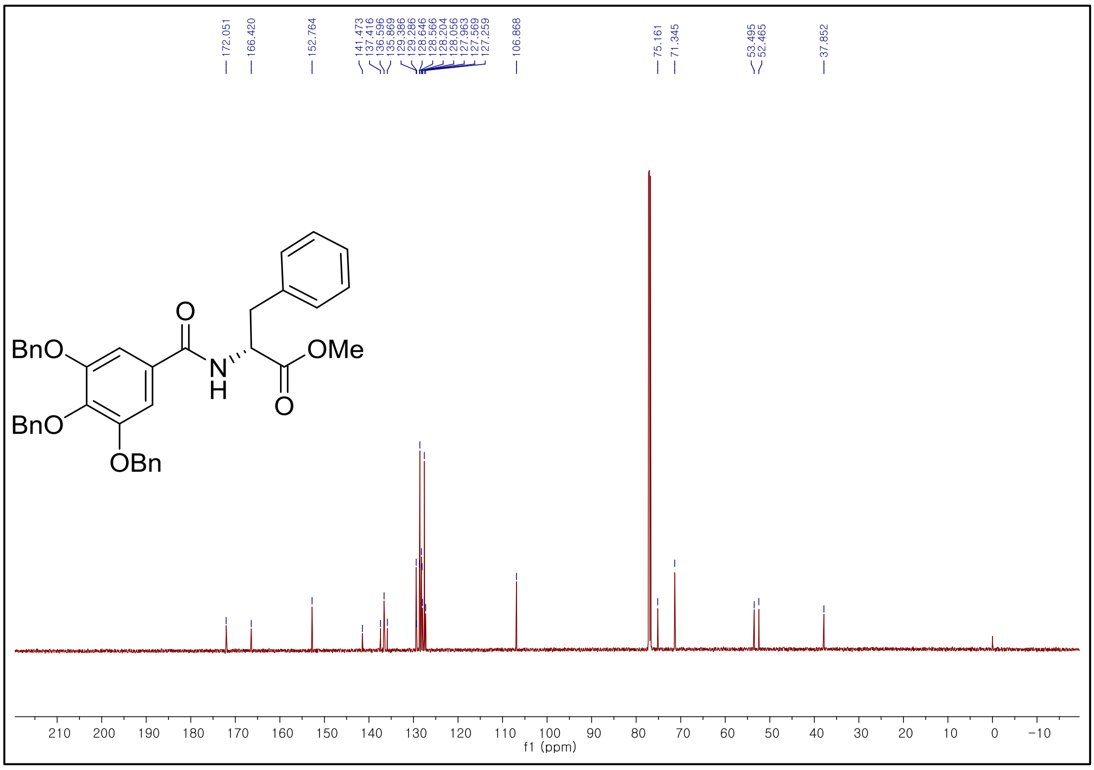

Figure S40. ^{13}C-NMR (150 MHz, $CDCl_3$) of **6c**.

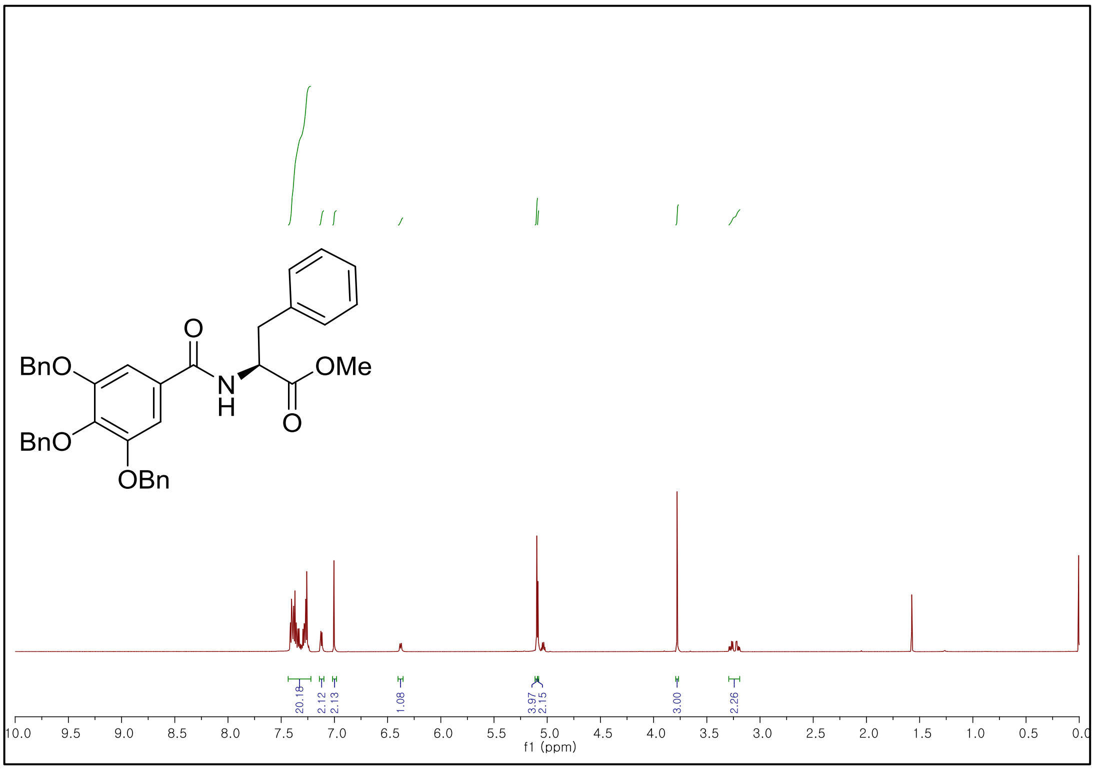

Figure S41. ^{1}H-NMR (600 MHz, $CDCl_3$) of **6d**.

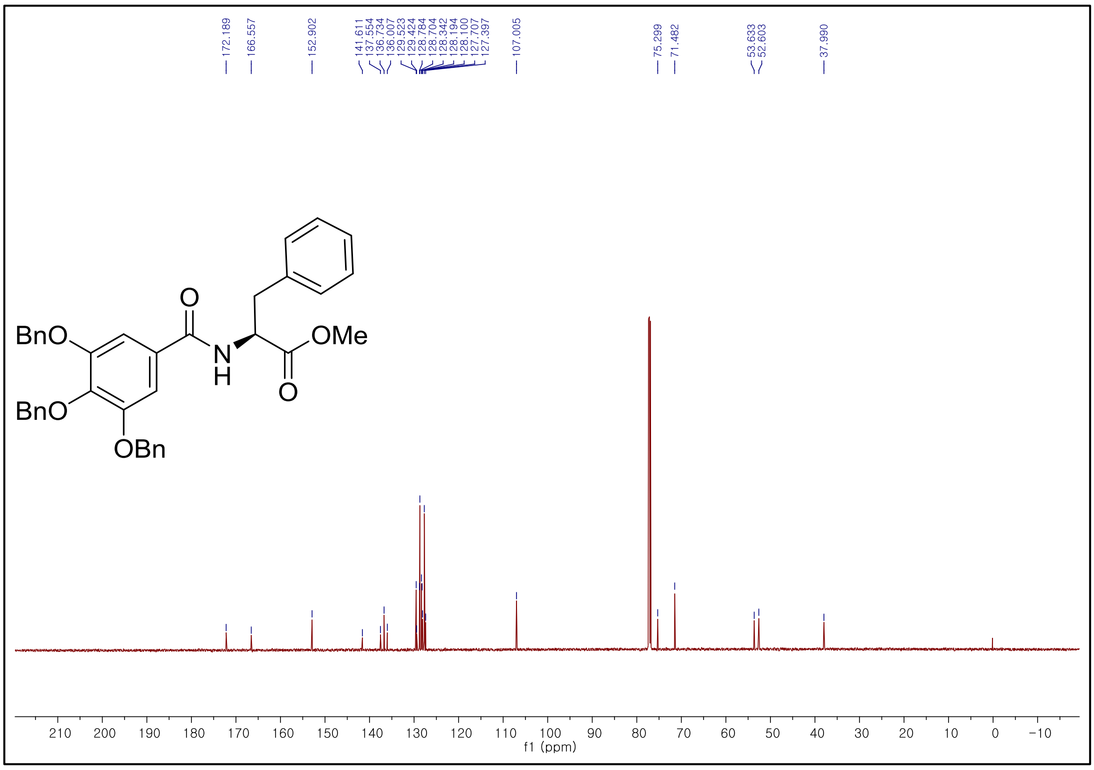

Figure S42. ^{13}C-NMR (150 MHz, $CDCl_3$) of **6d**.

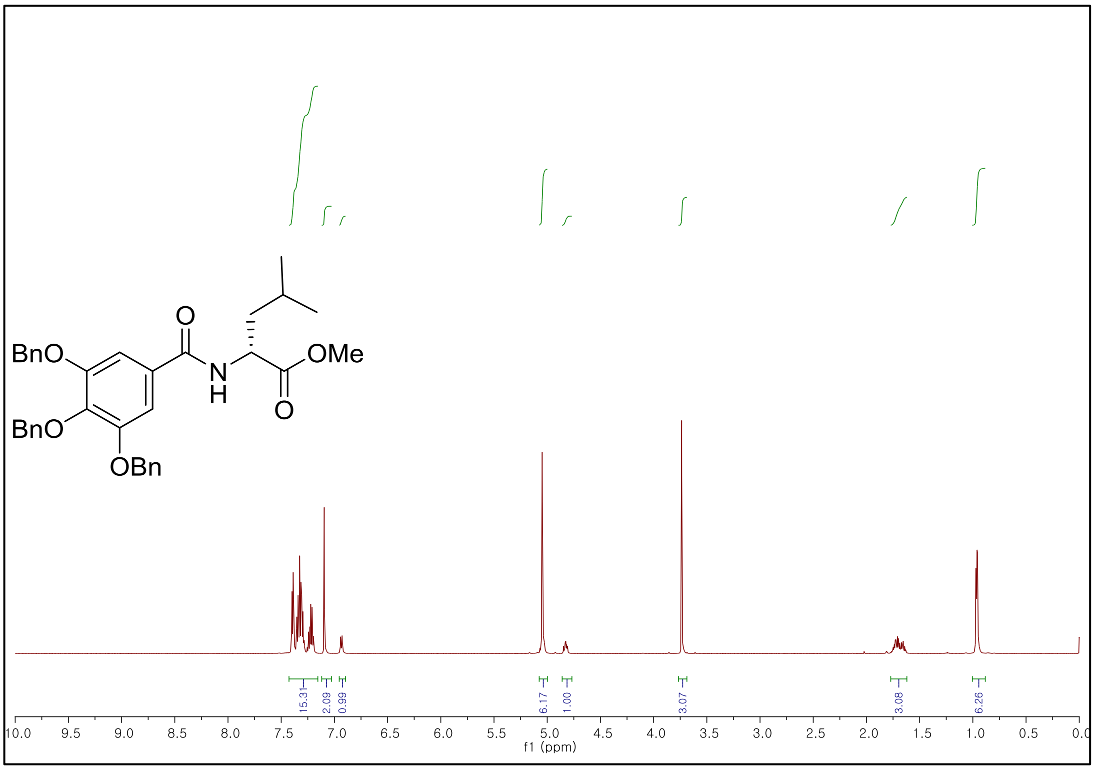

Figure S43. ^{1}H-NMR (600 MHz, $CDCl_3$) of **6e**.

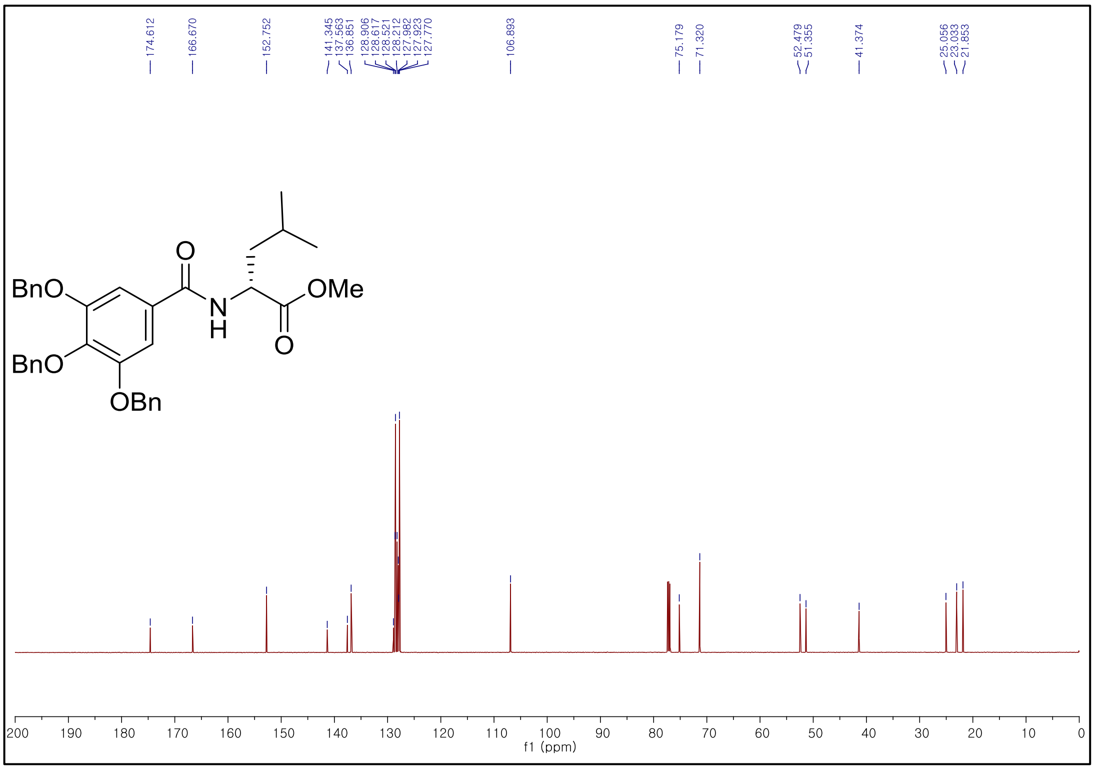

Figure S44. ^{13}C-NMR (150 MHz, $CDCl_3$) of **6e**.

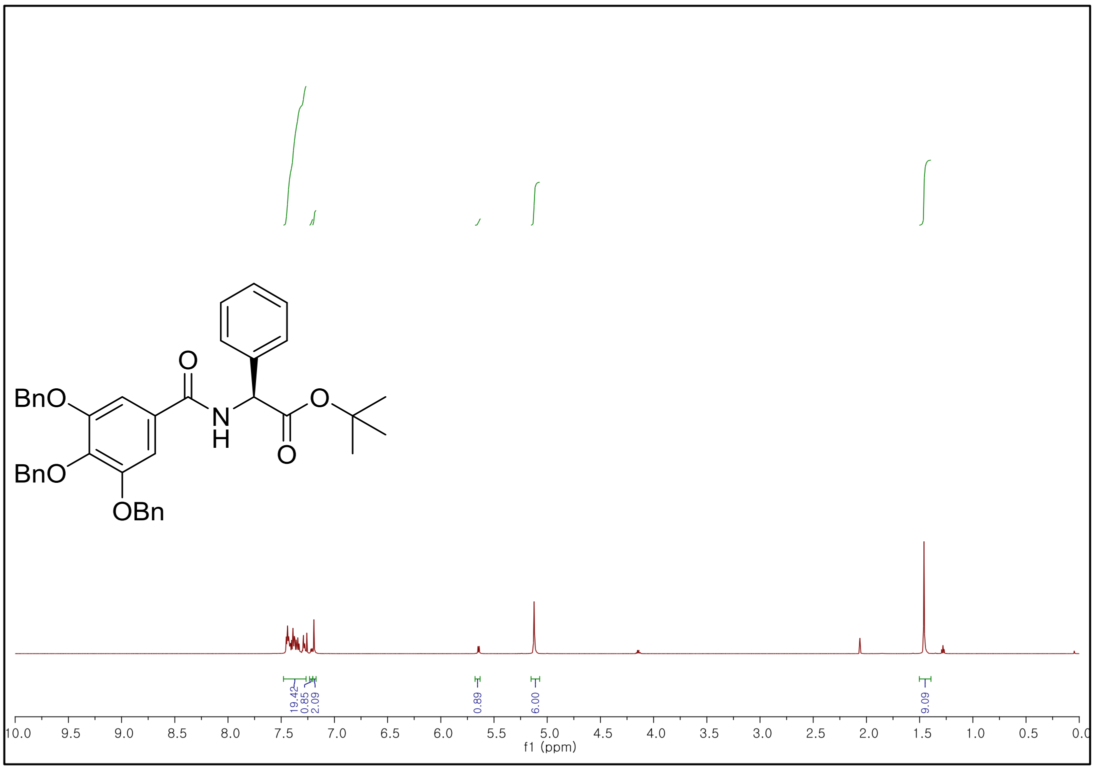

Figure S45. ^{1}H-NMR (600 MHz, $CDCl_3$) of **6f**.

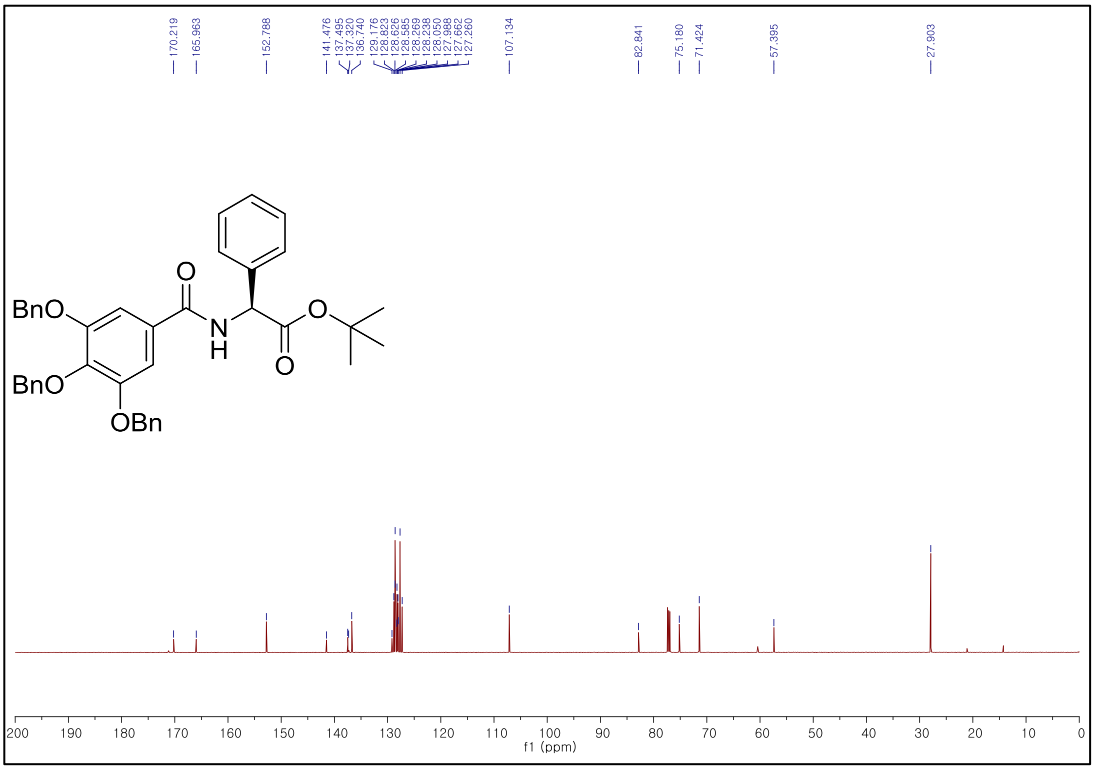

Figure S46. ^{13}C-NMR (150 MHz, $CDCl_3$) of **6f**.

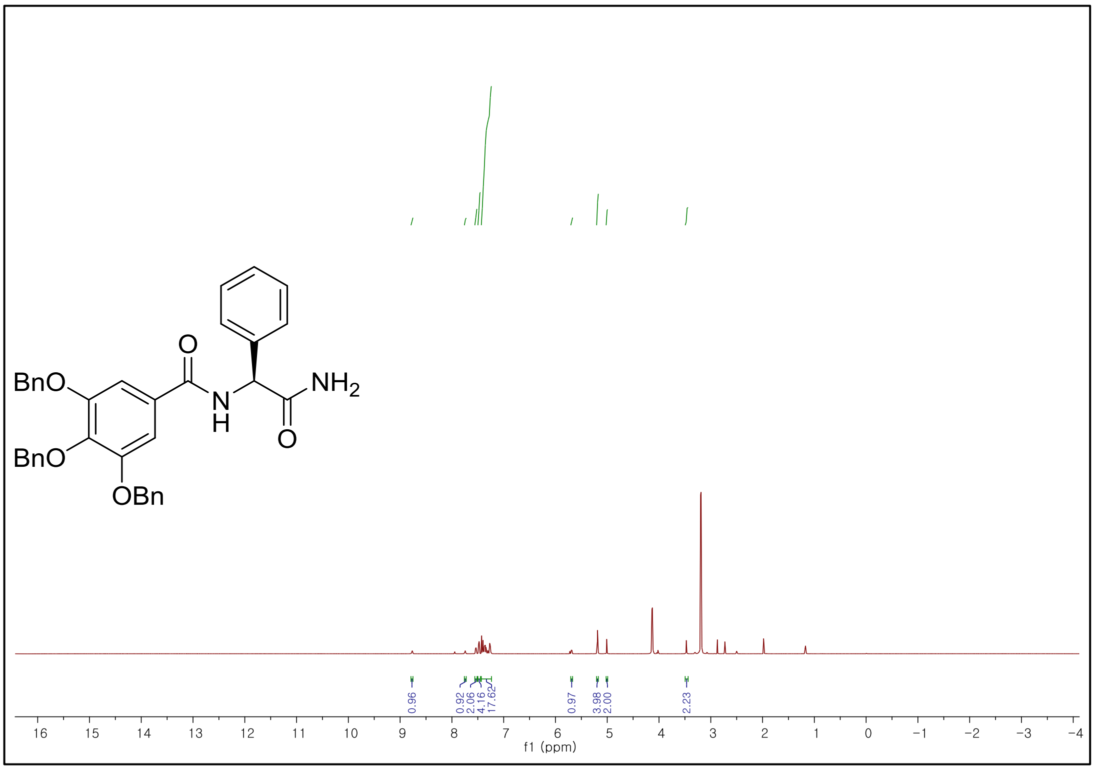

Figure S47. ^{1}H-NMR (600 MHz, DMSO-d$_6$) of **6g**.

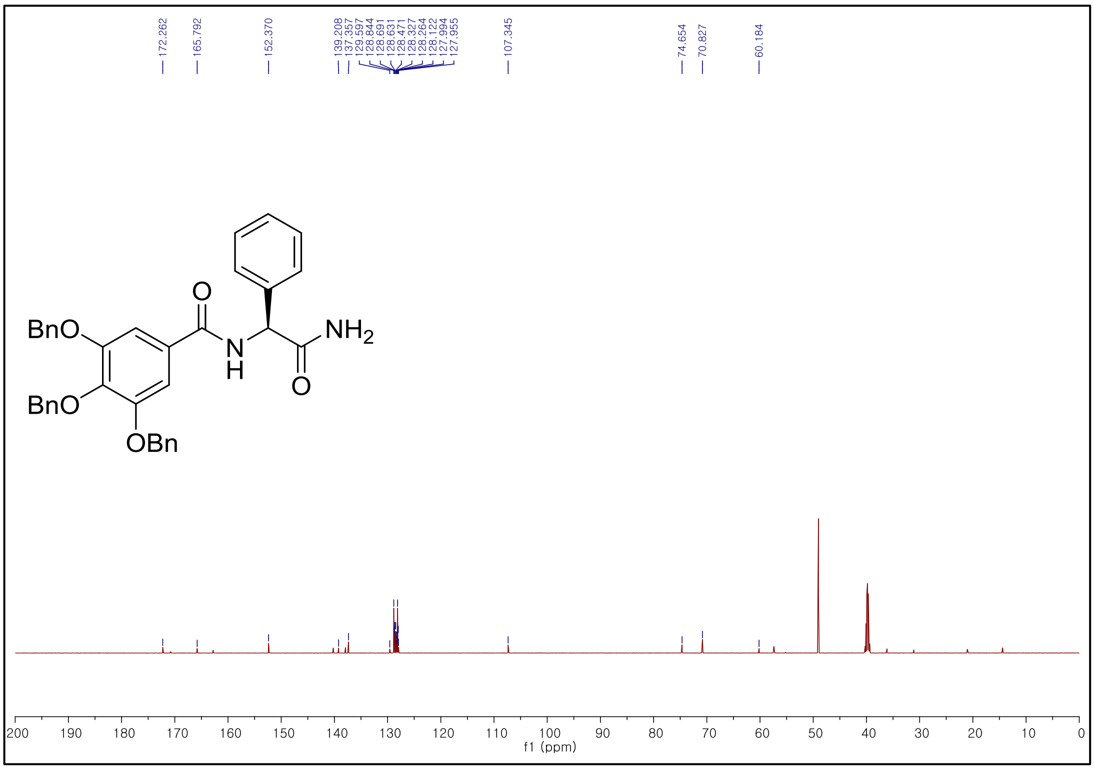

Figure S48. ^{13}C-NMR (150 MHz, DMSO-d$_6$) of **6g**.

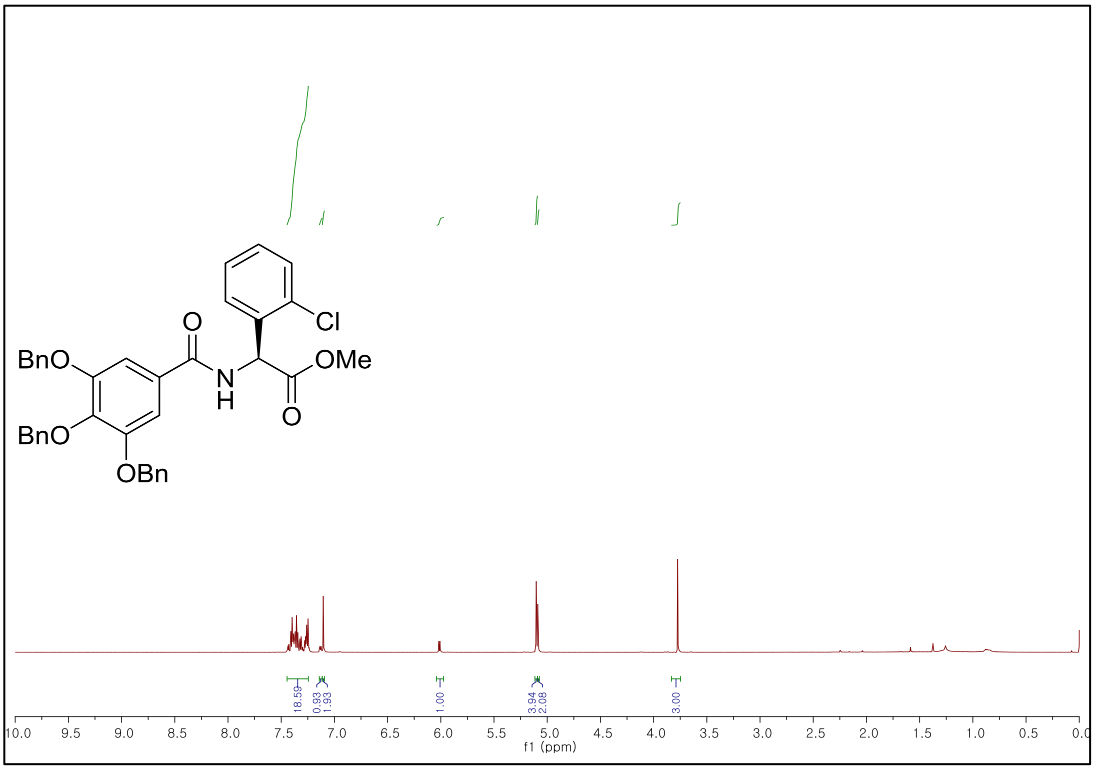

Figure S49. ^{1}H-NMR (600 MHz, $CDCl_3$) of **6h**.

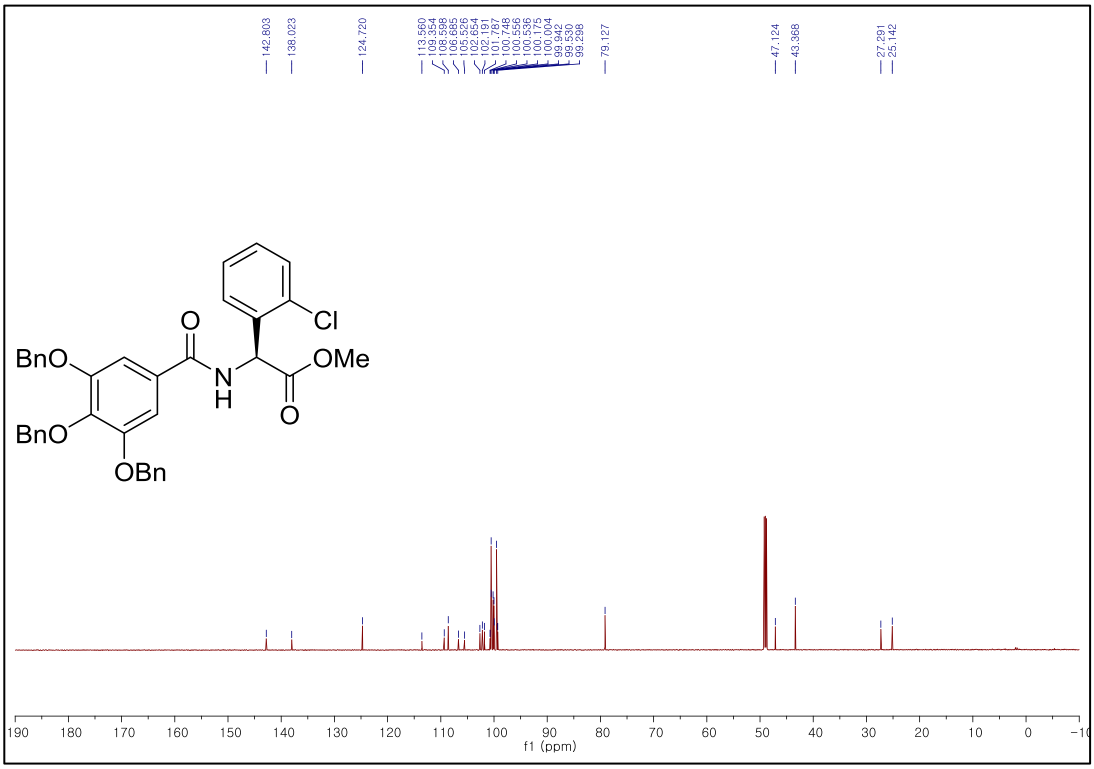

Figure S50. ^{13}C-NMR (150 MHz, $CDCl_3$) of **6h**.

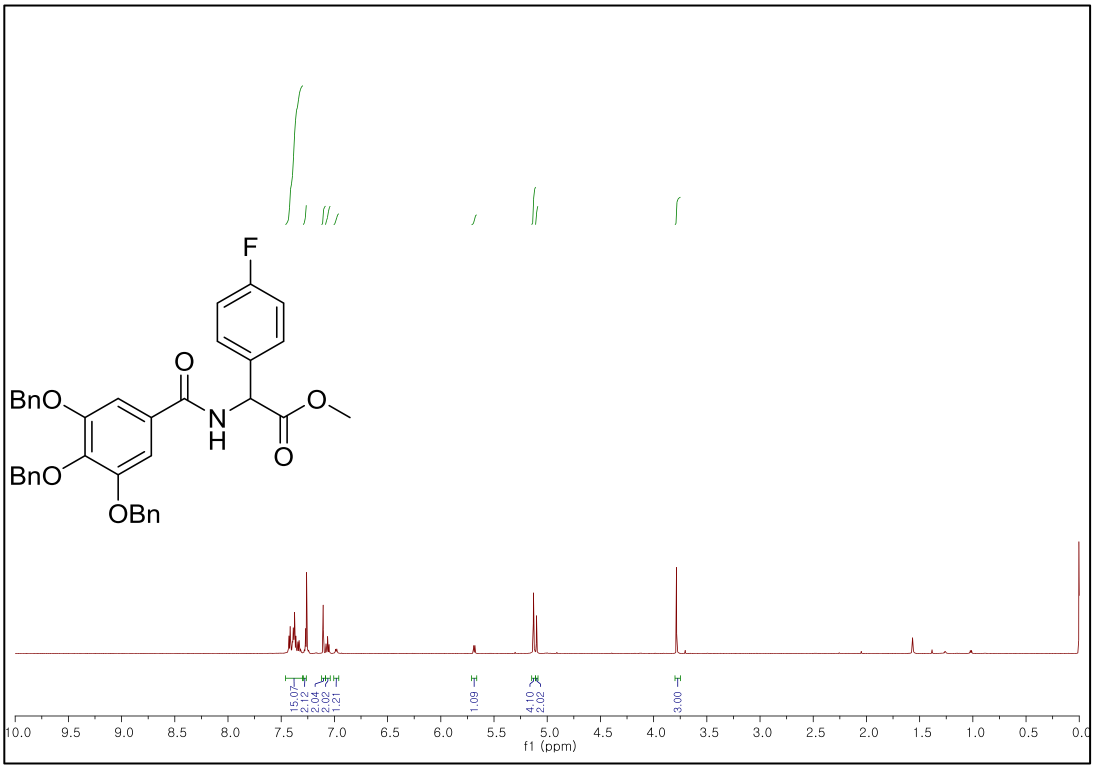

Figure S51. ^{1}H-NMR (600 MHz, $CDCl_3$) of **6i**.

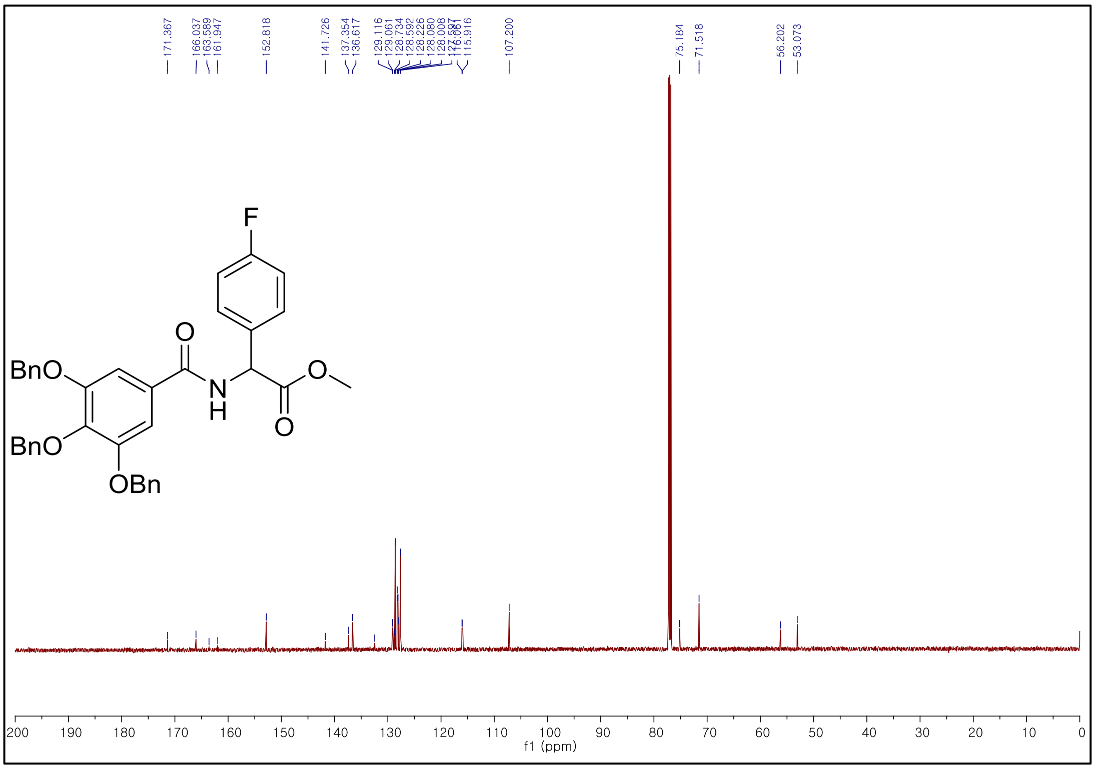

Figure S52. ^{13}C-NMR (150 MHz, $CDCl_3$) of **6i**.
